# Functional brain activity in cancer-related cognitive impairment: a scoping review of EEG studies

**DOI:** 10.3389/fnhum.2026.1851560

**Published:** 2026-10-15

**Authors:** Barbara Johanne Thomas Nordhjem, Laura Maria Toftekær Rothschild, Clotilde Rose Honoré, Anders Thyge Steen Larsen, Martin Kaj Fridh, Lisa Lyngsie Hjalgrim

**Affiliations:** 1 Department of Pediatrics and Adolescent Medicine, Copenhagen University Hospital - Rigshospitalet, Copenhagen, Denmark; 2 UCSF - Centre for Health Research, Copenhagen University Hospital - Rigshospitalet, Copenhagen, Denmark

**Keywords:** cancer survivorship, cancer-related cognitive impairment, cognition, electroencephalography, event-related potentials, quantitative EEG

## Abstract

Cancer-related cognitive impairment (CRCI) is reported across the cancer trajectory, yet its neurobiological basis remains incompletely understood. Electroencephalography (EEG) provides a temporally precise method for assessing brain activity at rest and during cognitive processing, but its application in CRCI research has not been systematically mapped. This scoping review characterized how EEG has been used to study cognitive functioning in people with cancer, including populations studied, EEG paradigms and analytic approaches employed, and cognitive outcomes assessed. Following PRISMA-ScR guidance, we searched MEDLINE, PsycINFO, and Web of Science from inception to February 2025 for studies using EEG to examine cognitive functioning. Sixty articles were included. Most were small and cross-sectional, with publication output increasing markedly after 2015. Task-based event-related potentials, particularly from oddball and attention tasks, were used most frequently, whereas quantitative EEG approaches, including spectral power and connectivity analyses, were less common. In populations with cancers outside the central nervous system (CNS), longer P300 latency and lower amplitude were common, but opposite and null findings also occurred. Evidence from primary CNS and other intracranial tumor populations was sparse. Across cancer contexts, quantitative EEG findings showed limited convergence across articles. Associations between EEG measures and cognitive outcomes were inconsistent and often not tested directly. Overall, EEG provides a complementary approach to investigating cognition in people with cancer, but the evidence remains methodologically heterogeneous. Larger longitudinal studies, more consistent cognitive outcome selection, and closer integration of neural and cognitive measures are needed to establish reproducible findings and evaluate their potential clinical utility.

**Systematic review registration:**

https://www.crd.york.ac.uk/PROSPERO/view/CRD420251084951.

## 1 Introduction

Cancer-related cognitive impairment (CRCI) is frequently reported in children, adolescents, and adults with cancer, both during active treatment and into long-term survivorship (Ahles and Root, 2018; Lange et al., 2019). Commonly affected domains include attention and concentration, learning and memory, processing speed, and executive functions such as working memory, cognitive flexibility, and planning. However, the pattern, severity, and course of cognitive difficulties vary substantially across individuals and cancer populations (Lange et al., 2019; Sleurs et al., 2022).

An important distinction can be drawn between patients with central nervous system (CNS) or other intracranial tumors and those with non-CNS cancers. In patients with intracranial tumors, cognition may be affected by tumor location, infiltration, and growth, tumor-related complications such as seizures or increased intracranial pressure, and the effects of surgery and brain-directed treatment (Taphoorn and Klein, 2004). In non-CNS cancers, cognitive difficulties typically cannot be attributed to a single focal brain lesion and have been associated with systemic chemotherapy, endocrine therapy, and targeted treatments, although cognitive impairment may also be detectable before treatment begins (Wefel et al., 2015; Joly et al., 2019; Scriney et al., 2026). Across populations, vulnerability is further influenced by age and developmental stage, pretreatment cognitive reserve, comorbidities, treatment intensity, and time since treatment (McDonald et al., 2010; Krull et al., 2018). In childhood cancer, younger age at diagnosis and exposure to cranial irradiation or neurotoxic systemic or intrathecal treatments are particularly important risk factors, reflecting the vulnerability of the developing brain (Krull et al., 2018).

CRCI findings also depend on how cognition is assessed. Standardized neuropsychological tests, behavioral performance during experimental tasks, and self-reported cognitive difficulties capture related but partly distinct aspects of cognitive functioning. Self-reported concerns are often weakly correlated with neuropsychological test performance and may reflect subtle changes or everyday difficulties that standardized tests do not detect. Fatigue, sleep disturbance, anxiety, and depression are more consistently associated with self-reported cognition than with objective performance, although they may also affect cognitive performance (Hutchinson et al., 2012; Bray et al., 2018; Oliva et al., 2024). Subjective and objective measures should therefore be regarded as complementary rather than interchangeable. Examining these outcomes alongside measures of neural function may help characterize the processes associated with CRCI.

Despite the identification of several clinical, treatment-related, and psychosocial risk factors, the neurobiological mechanisms underlying CRCI remain incompletely understood (Ahles and Root, 2018; Sleurs et al., 2022). Animal models of chemotherapy-related CRCI point to several interacting processes, including neuroinflammation and oxidative stress, reduced hippocampal neurogenesis and synaptic plasticity, and altered oligodendrocyte function and myelin maintenance. These changes have been associated with impaired hippocampal-dependent learning and memory, while myelin and synaptic abnormalities may also affect communication across broader neural networks (Nguyen and Ehrlich, 2020; John et al., 2021; Sekeres et al., 2021). However, preclinical studies include both tumor-free and tumor-bearing models and use varied treatment regimens. Their strong emphasis on hippocampal learning and memory may limit how well they capture the broader clinical and treatment context of CRCI.

Human neuroimaging studies of CRCI have primarily relied on magnetic resonance imaging (MRI), with a strong emphasis on adults with breast cancer (Horowitz et al., 2018; McDonald, 2021; Silverman et al., 2025). Structural MRI studies have reported reduced gray-matter volume or density in frontal and temporal regions, including the prefrontal cortex and hippocampus, as well as alterations in cortical thickness (McDonald et al., 2010; McDonald, 2021). Diffusion imaging studies have identified reduced white-matter microstructural integrity after treatment, including changes in frontal and temporal regions and major white-matter tracts (Deprez et al., 2012; McDonald, 2021). Some longitudinal studies have linked these changes to attention, processing speed, and memory. The course of these changes is variable, with partial recovery reported in some cohorts and persistent alterations in others (Billiet et al., 2018; McDonald, 2021).

Functional MRI studies suggest that CRCI may also involve altered recruitment and coordination of distributed neural systems. Task-based studies have shown both increased and decreased activation in frontal and parietal regions during attention and working-memory tasks, sometimes despite relatively preserved behavioral performance (McDonald et al., 2012; Silverman et al., 2025). Increased activation has been interpreted as compensatory or inefficient recruitment, whereas reduced activation may indicate insufficient recruitment, although these interpretations remain context dependent. Resting-state studies have identified altered connectivity involving frontoparietal control, default mode, and hippocampal networks, but the location and direction of findings vary across studies (Conti et al., 2025; Silverman et al., 2025). Overall, MRI findings point to a combination of regional, white-matter, and network-level changes, but their relationships with well-characterized cognitive outcomes remain inconsistent.

Electroencephalography (EEG) offers a complementary approach to investigating these functional changes. EEG records cortical electrophysiological activity with millisecond temporal resolution and can be acquired noninvasively using comparatively portable and low-cost equipment, facilitating repeated assessment in clinical settings. Although scalp EEG has less anatomical specificity than MRI, it can characterize the timing and coordination of neural activity at rest and during cognitive processing. The information obtained depends strongly on the recording condition, paradigm, and analytic approach. Table 1 provides an overview of key design dimensions relevant to CRCI EEG research, including resting-state versus task-based recording and event-related potential (ERP), spectral, connectivity, and network-based analyses.

**Table 1:** EEG recording conditions, signal types and typical analysis strategies relevant to CRCI research.

Recording condition	Derived feature type	Typical metrics and analysis strategy
*Task-based EEG* - Recorded during structured paradigms - Examples: oddball/deviance, go/no-go, flanker/Attention Network Test (ANT-R), and working memory tasks	*Event-related potentials (ERPs)* - Time-locked voltage responses to discrete events, such as target versus standard stimuli in oddball paradigms - Components labeled by polarity and latency, such as N100, N200, and P300	*Component-based analysis* - Predefined time windows - Peak or mean amplitude - Latency estimation Primary outputs: amplitude and latency differences between conditions or groups
*Resting-state EEG* - Eyes open or eyes closed - No explicit task	*Quantitative EEG (qEEG)* - Spectral power - Peak frequency - Time–frequency measures - Functional connectivity measures - Microstate parameters	*Spectral, connectivity, and microstate analyses* - Absolute or relative band power - Peak frequency estimation - Time–frequency decomposition - Coherence, phase-based metrics, or network indices - Aperiodic (1/f) parameters, such as spectral exponent and offset - Microstate duration, occurrence, coverage, and transition measures Primary outputs: oscillatory and aperiodic spectral characteristics, connectivity patterns, and temporal microstate properties

This table provides a conceptual overview of common EEG recording conditions, feature types, and analytic strategies relevant to CRCI research. These dimensions are not mutually exclusive: qEEG methods can be applied to resting-state and task-based recordings, whereas ERPs require events to which EEG activity can be time-locked (Polich, 2007; Luck, 2014; Fries, 2015; Herrmann et al., 2016; Donoghue et al., 2020).

Resting-state recordings are commonly analyzed using quantitative EEG (qEEG) methods applied to spectral power, signal complexity, functional connectivity, and network organization (Popa et al., 2020). Brain oscillations reflect coordinated activity within neuronal populations and have been investigated in relation to cognition and cognitive disorders (Başar and Güntekin, 2008; Babiloni et al., 2011). Conventional analyses divide oscillatory activity into frequency bands such as delta, theta, alpha, beta, and gamma. However, band-limited power findings frequently overlap across disorders and vary with recording and analytic methods, limiting their diagnostic specificity (Newson and Thiagarajan, 2019). More recent approaches distinguish oscillatory peaks from aperiodic activity, the broadband, approximately 1/f-shaped component of the EEG power spectrum (Donoghue et al., 2020). Aperiodic measures are increasingly being investigated as potential clinical markers, although methods and findings remain heterogeneous (Donoghue, 2025; Hemmerich et al., 2026).

Task-based EEG can be examined using spectral and connectivity measures but frequently focuses on ERPs. Averaging activity time-locked to discrete stimuli, responses, or other behavioral events can distinguish successive stages of processing within a few hundred milliseconds, including sensory encoding, attentional selection, stimulus evaluation, and response preparation. ERP amplitudes and latencies can therefore provide information about the strength and timing of cognitive processes, although their interpretation depends on the component and paradigm. For example, P300 amplitude is commonly interpreted in relation to attentional-resource allocation and context updating, whereas P300 latency provides an index of stimulus-evaluation speed (Polich, 2007).

Despite these potential contributions, EEG research on cognition in people with cancer has not been mapped systematically. It remains unclear which cancer populations and stages of the cancer trajectory have been studied, which EEG paradigms and analytic approaches have been used, and how EEG measures have been related to behavioral task performance, standardized neuropsychological tests, and self-reported cognitive or symptom measures. This methodological and outcome heterogeneity complicates comparisons across studies and limits understanding of the current evidence.

The aim of this scoping review was to systematically map studies assessing cognitive functioning in relation to brain activity measured with EEG in people with cancer across the cancer trajectory. Specifically, we sought to (1) identify the cancer populations and treatment phases studied; (2) characterize the EEG methods used, including resting-state and task-based paradigms and their derived measures; (3) summarize the cognitive domains and assessment approaches used; (4) synthesize reported EEG findings and their relationships with behavioral task performance, neuropsychological test results, and self-reported cognitive or related symptom outcomes; and (5) identify recurring methodological limitations and gaps relevant to future research.

## 2 Methods

### 2.1 Reporting guideline and protocol registration

This review was conducted and reported in accordance with the Preferred Reporting Items for Systematic Reviews and Meta-Analyses extension for Scoping Reviews (PRISMA-ScR) guidelines (Tricco et al., 2018). The completed PRISMA-ScR checklist is provided in Supplementary material 1. A protocol was developed *a priori* and registered in PROSPERO as a systematic review (CRD420251084951). During the screening and initial data charting, substantial heterogeneity was identified in EEG paradigms, analytic approaches, and cognitive outcomes. We therefore adopted a scoping review approach to map the available evidence and identify research gaps.

### 2.2 Eligibility criteria

Eligibility criteria were defined using the Population–Concept–Context (PCC) framework.

*Population*. We included studies of human participants of any age and sex diagnosed with cancer, either during active treatment or after completion of treatment.

*Concept*. Studies were eligible if they reported a quantified scalp EEG outcome used to assess cognition, either with a cognitive paradigm or alongside a measure of cognitive functioning in people with cancer. Cognitive outcomes primarily included: (i) standardized neuropsychological tests, (ii) self-reported cognitive measures, and (iii) behavioral performance during experimental tasks. During data charting, one study using a clinician-rated scale explicitly to classify cognitive impairment was also retained. EEG outcomes were required to be reported as quantified parameters such as ERP amplitude or latency, spectral power, peak frequency, aperiodic parameters, connectivity or coherence measures, or microstate parameters. EEG and cognitive outcomes could be reported separately within the same study; a direct statistical association between them was not required.

*Context*. Studies conducted in any setting (hospital, outpatient, community, academic research settings) and in any country were eligible.

*Study designs*. We included original, peer-reviewed empirical studies using observational or experimental designs. Eligible designs included cross-sectional studies, longitudinal designs, pre-post (intervention-based) studies, and randomized controlled trials. Studies could involve prospectively or retrospectively identified cancer cohorts, if EEG and cognitive outcomes were systematically assessed and reported. Studies had to be published in English.

*Exclusion criteria*. We excluded: (1) case reports, case series, and any studies with fewer than five participants; (2) reviews, meta-analyses, editorials/commentaries, protocols, and conference abstracts; (3) animal studies; (4) studies not published in English; (5) studies using only methods other than scalp EEG (e.g., functional MRI [fMRI], magnetoencephalography [MEG], or invasive recordings); (6) studies in which EEG was used solely for routine clinical interpretation such as seizure detection or background assessment, or in which EEG results were limited to qualitative classifications (e.g., “normal” vs. “abnormal,” slowing grades, epileptiform activity); (7) studies that did not report ERP or qEEG parameters (e.g., ERP latencies/amplitudes, spectral power, peak frequency, connectivity); (8) studies reporting only sensory evoked potentials (e.g., visual, brainstem auditory, or somatosensory evoked potentials); and (9) studies focused on cancer predisposition syndromes (e.g., neurofibromatosis type 1) rather than cancer diagnosis or treatment.

*Comparator information*. Studies were not required to include a specific comparator (e.g., healthy controls or pre-treatment baseline) to be eligible for inclusion. Where comparator data were available, the type of comparison was charted and summarized descriptively.

The main eligibility criteria were defined *a priori*. Retention of the clinician-rated measure was treated as a *post hoc* clarification during data charting.

### 2.3 Information sources and search strategy

We searched the following electronic databases: MEDLINE (Ovid), PsycINFO (APA PsycNet), and Web of Science Core Collection (Clarivate) from database inception to 12 February 2025. The initial search was conducted on 26 July 2023 and updated on 12 February 2025. The search strategy combined three concept blocks: (1) cancer/neoplasm terms, (2) EEG and related electrophysiological terms, and (3) cognition/cognitive function terms. Controlled vocabulary (e.g., Medical Subject Headings [MeSH]) and free-text keywords were combined using Boolean operators (AND/OR) and truncation where appropriate.

An abridged example of the MEDLINE (Ovid) search was:

1. exp Neoplasms/ OR cancer*.ti,ab,kw. OR leuk?emia.ti,ab,kw. OR malign*.ti,ab,kw. OR chemo*.ti,ab,kw.
2. exp Electroencephalography/ OR Event-Related Potentials, P300/ OR Evoked Potentials/ OR electroencephalogra*.ti,ab,kw. OR EEG.ti,ab,kw. OR qEEG.ti,ab,kw.
3. Cognition/ OR cognit*.ti,ab,kw. OR “chemo brain”.ti,ab,kw.
4. 1 AND 2 AND 3

The complete search strategies for all databases are provided in Supplementary material 2. In addition, reference lists of included studies were screened to identify further eligible articles.

### 2.4 Study selection

Records were imported into Covidence (Veritas Health Innovation, Melbourne, Australia) for deduplication and screening. Titles and abstracts were screened independently by two reviewers. Full texts were retrieved for potentially eligible studies and assessed independently by two reviewers against the eligibility criteria. Disagreements were resolved through discussion and consensus; if consensus could not be reached, a third reviewer was involved.

### 2.5 Data charting

Data were charted by one reviewer and independently checked by a second reviewer. Discrepancies in charted data were resolved by discussion and consensus.

For each included study, we charted:

1. Bibliographic information (first author, year of publication, country)
2. Study design (cross-sectional/longitudinal; observational/experimental; prospective/retrospective)
3. Participant characteristics (sample size, age, sex distribution)
4. Cancer characteristics (cancer type; treatment stage [on-treatment vs. post-treatment]; time since treatment where reported)
5. Treatment exposure(s) (e.g., chemotherapy, radiotherapy, immunotherapy, targeted therapy, CNS-directed treatment, intrathecal chemotherapy), where reported
6. Comparator characteristics where applicable (e.g., healthy controls, baseline measures, normative data, subgroup comparisons)
7. Cognitive outcomes (tests/questionnaires/tasks used; cognitive domains targeted; key results as reported by authors)
8. EEG methodology (resting-state vs. task-based; recording parameters if reported; preprocessing; analytic approach; EEG outcomes reported)
9. EEG findings and reported statistical associations between EEG measures and cognitive or related outcomes.

We did not contact authors for additional information.

### 2.6 Synthesis of results

Included articles were summarized and grouped according to key methodological and clinical characteristics, including EEG paradigm (resting-state vs. task-based), EEG analytic approach (e.g., quantitative EEG measures such as spectral power or connectivity; event-related potentials), cognitive domain(s) assessed, cancer type, and treatment stage (during treatment vs. survivorship). Bibliometric characteristics, including year of publication, geographic distribution, and sample size, were also extracted and summarized to characterize trends in the literature over time.

Findings were synthesized narratively and descriptively, in accordance with scoping review methodology. The unit of synthesis was the article; because some records represented fully or partially overlapping cohorts, publication counts were not interpreted as independent replications. Descriptive analyses and figures were produced using R version 4.4.1 (R Core Team, 2024).

For the cross-study narrative synthesis, publications were assigned to six categories that were not mutually exclusive: (1) P300-focused and oddball/deviance ERPs; (2) other task-related ERPs; (3) resting-state and sleep spectral EEG; (4) task-related spectral and aperiodic EEG; (5) connectivity and network measures; and (6) quantified background EEG in acute neurotoxicity. Assignment was based on the EEG feature supporting each finding, so a publication could contribute to more than one category.

Within each category, findings were stratified as relating to primary CNS or other intracranial tumor populations, non-CNS cancer populations, or explicitly mixed populations. Mixed CNS/non-CNS cancer samples and mixed tumor/traumatic-brain-injury samples were labeled separately rather than pooled into either stratum. This distinction was used to separate EEG changes potentially reflecting tumor location, neurological burden, surgery, seizures, or CNS-directed treatment from findings in cancers outside the CNS.

EEG-cognition evidence was coded as a direct association only when a statistical relationship was reported between an EEG measure and a standardized cognitive test, subjective cognitive or symptom measure, or behavioral task-performance outcome. Task-related EEG modulation, simultaneous group differences, and parallel longitudinal changes in EEG and cognition were not classified as direct associations. Separate publications reporting distinct analyses of fully or partially overlapping cohorts were retained and flagged in the synthesis. Exact duplicate records were removed.

In line with the mapping aim of this scoping review, we did not conduct a formal risk-of-bias assessment. Common methodological limitations were instead summarized descriptively at the literature level.

### 2.7 Classification of EEG approaches and paradigms

EEG methods were classified descriptively to support systematic mapping of approaches rather than inferential comparison. Articles were first categorized as reporting ERPs only, qEEG measures only, or both. Recording contexts were charted separately and could include task-based, resting-state, sleep, or multiple contexts.

Each task-based article was assigned one broad paradigm class: oddball/deviance, attention or executive control, memory, affective or emotional processing, or another task-based paradigm. Classification was based on the task domain rather than the ERP component alone. When an article included multiple paradigms, classification reflected the paradigm most central to the reported cognitive analysis.

qEEG measures were charted according to the derived measures reported, including spectral power and peak frequency, aperiodic parameters, connectivity, coherence or phase-based measures, network metrics, microstate parameters, and related quantitative indices. For descriptive summaries, each qEEG article was assigned one primary analytic family and one primary qEEG recording context. Primary analytic families were summarized as spectral measures, connectivity or phase-based measures, and aperiodic measures. When multiple families or contexts were reported, classification reflected the principal qEEG analysis; context was classified as unclear when it could not be determined from the article.

Given the wide variability in task design, EEG acquisition parameters, analytic methods, and reported outcomes, these classifications reflect broad conceptual and methodological orientations and do not imply equivalence or direct comparability of EEG measures across studies.

### 2.8 Classification of cognitive and related outcome measures

Cognitive and related outcome measures were organized into the following domains:

- Global cognition screeners: Brief instruments assessing overall status (e.g., Mini-Mental State Examination [MMSE], Montreal Cognitive Assessment [MoCA], Addenbrooke’s Cognitive Examination [ACE]).
- General cognitive ability: IQ-oriented batteries (e.g., Wechsler Adult Intelligence Scale [WAIS], Wechsler Intelligence Scale for Children [WISC], Wechsler Preschool and Primary Scale of Intelligence [WPPSI]).
- Neuropsychological tests: Attention/executive (e.g., Trail Making Test, Stroop Test, Continuous Performance Test [CPT]); processing speed (e.g., Symbol Search, Coding); memory, learning and working memory (e.g., Rey Auditory Verbal Learning Test [RAVLT], California Verbal Learning Test [CVLT], Hopkins Verbal Learning Test [HVLT], Digit Span, Corsi Block-Tapping Test); language/academic (e.g., naming, fluency, reading); visuospatial/reasoning (e.g., Rey–Osterrieth Complex Figure, Raven’s Progressive Matrices).
- Subjective measures: Self-reported complaints (e.g., Functional Assessment of Cancer Therapy – Cognitive Function [FACT-Cog], Patient Assessment of Own Functioning Inventory [PAOFI]) and symptom-related patient-reported outcomes (e.g., fatigue, mood, health-related quality of life). One study used the clinician-rated Assessment of Symptoms in Gerontopsychiatry to classify the severity of cognitive impairment (Olbrich et al., 1986). This measure was charted separately and was not grouped with self-reported measures.
- Experimental tasks: Behavioral performance from cognitive/psychophysiological paradigms (e.g., oddball, Go/No-Go, flanker; reaction time and accuracy). A study was included in this category only when behavioral performance, such as reaction time, accuracy, or errors, was reported; use of a cognitive task solely to elicit EEG activity was not sufficient.

Articles could contribute to multiple categories. For the descriptive figure, measures were summarized under three overarching categories: standardized cognitive tests, subjective measures, and behavioral task performance measures.

## 3 Results

### 3.1 Literature search and study selection

The database search identified 3,577 records. After removal of duplicates (1,903), 1,674 records were screened by title and abstract, of which 1,549 were excluded. Of the 125 reports sought for retrieval, three could not be obtained and 122 articles were assessed for eligibility. Following full-text assessment, 62 reports were excluded, most commonly because they reported clinical EEG only, used an ineligible EEG method, or had an ineligible study design or cognitive outcome.

A total of 60 articles met the eligibility criteria and were included in the scoping review. The article-selection process and reasons for exclusion are summarized in Figure 1, and the characteristics of the included articles are provided in Supplementary material 3.

**Figure 1:**
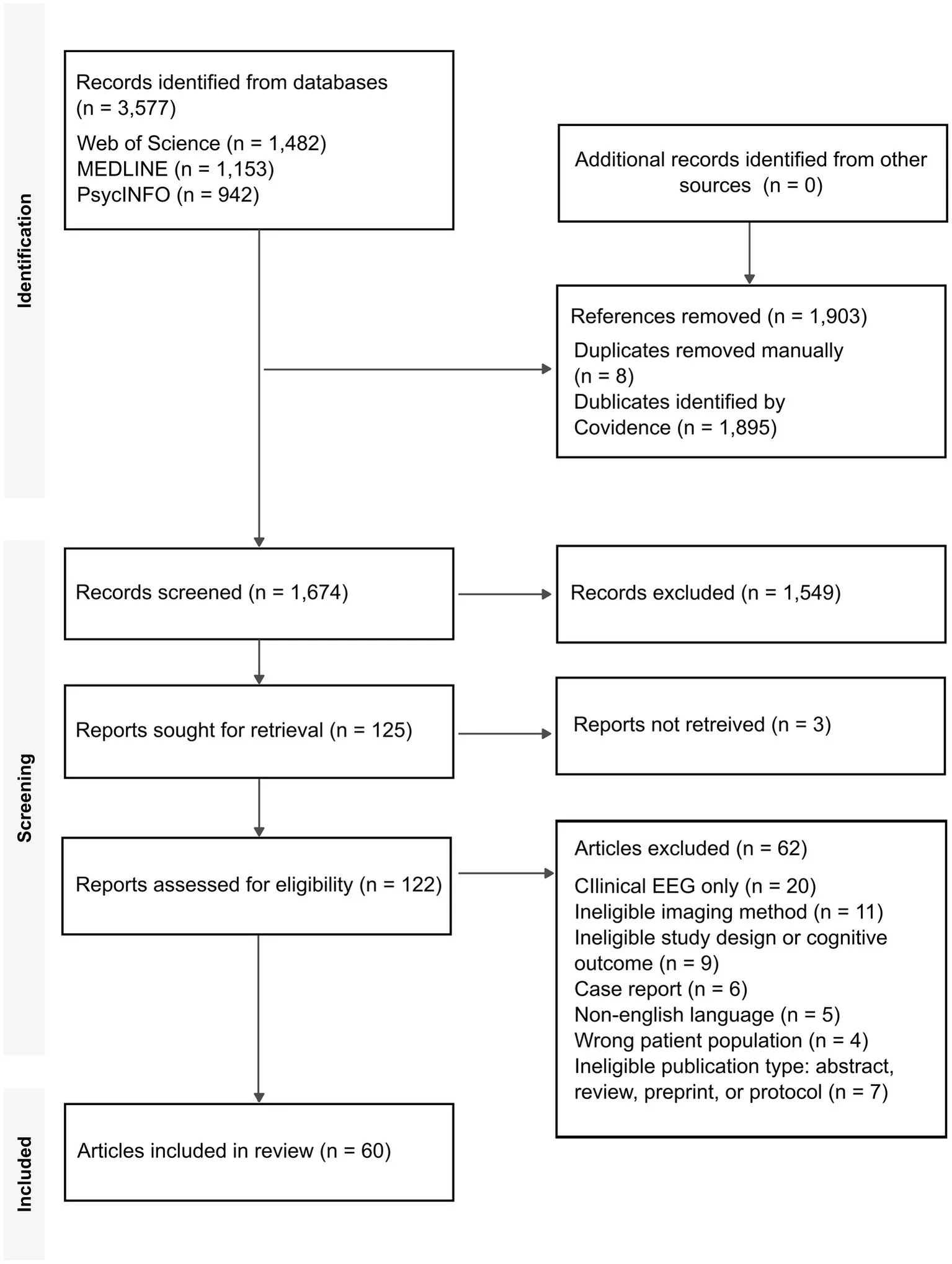
PRISMA flow diagram of article selection. Records were identified through MEDLINE, PsycINFO, and Web of Science. The searches were last updated on 12 February 2025.

### 3.2 Characteristics of included articles

#### 3.2.1 Publication year and geographic distribution

The 60 included articles were published between 1981 and 2025. Of these, 48 examined non-CNS cancers, 9 examined primary CNS/intracranial tumors, and 3 included mixed/other samples. Figure 2 presents complete 5-year publication intervals through 2024. The two articles published in 2025 were not plotted because the 2025–2029 interval was incomplete. Publication output was low and variable through 2009, increased in 2010–2014, and rose further thereafter. This later growth was driven mainly by non-CNS cancer research, which increased markedly from 2015 onward. Primary CNS/intracranial tumor research remained sparse: apart from 1 article published in 1985–1989, the remaining 8 were published from 2010 onward, including 5 in 2020–2024. All 3 mixed/other articles were published in 2010–2014.

**Figure 2:**
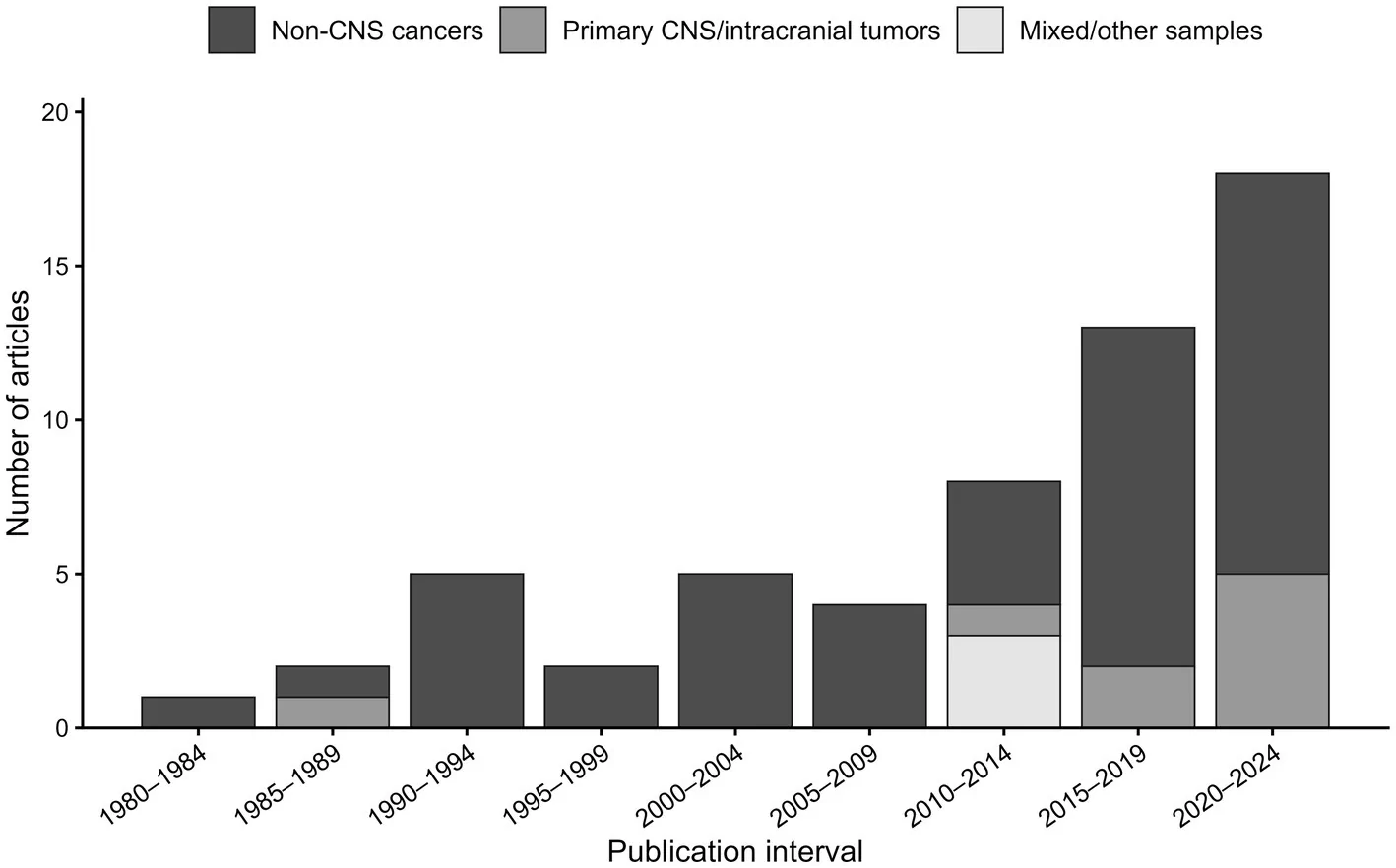
Included articles by publication interval and cancer context. Bars show the number of articles published within each 5-year interval, stratified by cancer context. The two articles published in 2025 were not plotted because the 2025–2029 interval was incomplete. Mixed/other samples included articles combining CNS and non-CNS cancers, primary CNS tumors and CNS metastases, or intracranial tumors and non-cancer brain injury. CNS, central nervous system.

Based on the locations of the study samples, 29 articles (48.3%) were conducted in Europe, 22 (36.7%) in North America, and 9 (15.0%) in Asia. No eligible articles were conducted exclusively in other world regions.

#### 3.2.2 Study design and sample size

Study design and sample size distributions are summarized in Figure 3. Of the 60 included articles, 33 (55.0%) were cross-sectional, 17 (28.3%) were longitudinal, 6 (10.0%) used pre-post or intervention-based designs, and 4 (6.7%) were randomized controlled trials.

**Figure 3:**
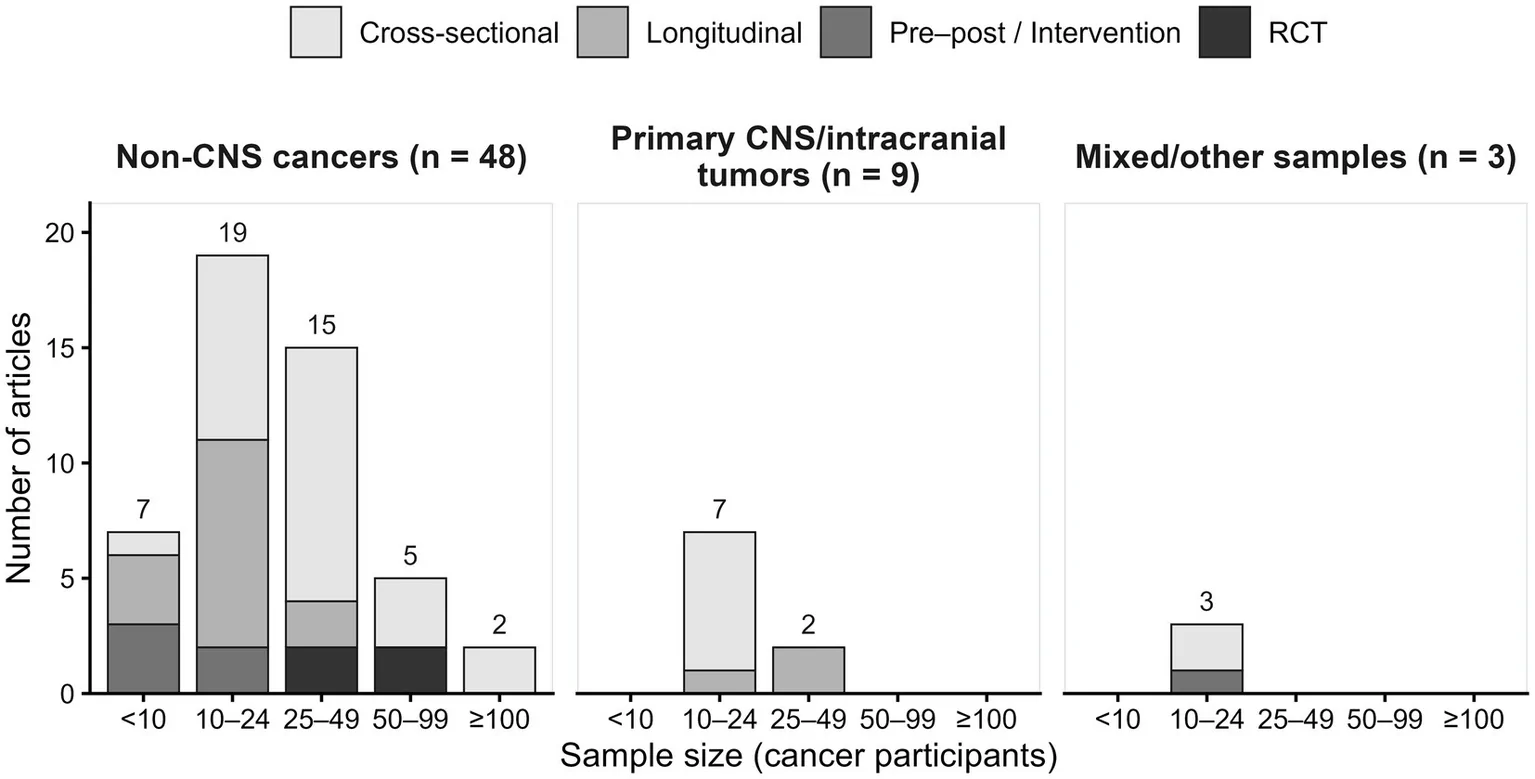
Study design and sample size by cancer context. Within each panel, bars represent the number of articles within each patient sample size category, stratified by study design. Sample size refers to the number of patient participants included in each article. Mixed/other samples included studies combining CNS and non-CNS cancers, primary CNS tumors and CNS metastases, or tumor-related and non-cancer brain injury.

Sample sizes were generally small. Of the 60 articles, 36 (60.0%) included fewer than 25 cancer patients, and 53 (88.3%) included fewer than 50. Only 7 articles (11.7%) included 50 or more cancer participants, including 2 (3.3%) with sample sizes of at least 100. All 9 primary CNS/intracranial tumor articles included fewer than 50 cancer participants: 7 included 10–24 and 2 included 25–49. All 3 mixed/other articles included 10–24 participants.

Cross-sectional articles were represented across all sample size categories but occurred most often in the 10–24 and 25–49 categories. Longitudinal articles also most commonly included fewer than 25 cancer participants. The primary CNS/intracranial tumor literature comprised 6 cross-sectional and 3 longitudinal articles. All pre-post/intervention articles included fewer than 25 cancer participants, whereas randomized controlled trials occurred only in articles enrolling 25 or more.

#### 3.2.3 Distribution of cancer types

Table 2 presents the distribution of mutually exclusive cancer categories. Breast cancer was the most frequently studied diagnosis (*n* = 21, 35.0%). Hematologic malignancies and primary CNS/intracranial tumors were the next most frequently represented categories (*n* = 8, 13.3% each). Other cancer types were less frequent, while 12 articles (20.0%) included mixed or broader cancer populations. Nine articles concerned childhood cancers: 4 examined one broad cancer category and 5 included mixed cancer populations.

**Table 2:** Cancer populations represented in the included articles.

Cancer population	Articles, *n*	%
**Studies within one broad cancer category**	**48**	**80.0**
Breast cancer	21	35.0
Primary CNS/intracranial tumors	8	13.3
Hematologic malignancies	8	13.3
Gastrointestinal cancers	4	6.7
Lung cancer	3	5.0
Head and neck cancer	2	3.3
Melanoma	1	1.7
Thyroid cancer	1	1.7
**Mixed or broader samples**	**12**	**20.0**
Mixed non-CNS cancers	8	13.3
Mixed primary and metastatic intracranial tumors	2	3.3
Mixed CNS and non-CNS cancers	1	1.7
Intracranial tumors and non-cancer brain injury	1	1.7

Categories are mutually exclusive, and each article was counted once. Diagnosis-based categories differ from the broader cancer-context groups used in Figures 2, 3. Nine articles concerned childhood cancers: 4 examined one broad cancer category and 5 included mixed cancer populations.

### 3.3 EEG recording contexts and analytical approaches

Across the 60 included articles, 35 (58.3%) included ERP measures only, 19 (31.7%) reported qEEG measures only, and 6 (10.0%) included both. Recording contexts were not mutually exclusive: task-based EEG paradigms were used in 47 articles (78.3%), resting-state in 18 (30.0%), and sleep EEG in 1 (1.7%).

#### 3.3.1 Task-based EEG paradigms and analytical approaches

Each task-based article was assigned one primary task domain for Figure 4A. Oddball/deviance paradigms (*n* = 20) and attention/executive control tasks (*n* = 21) were most frequently used. Memory paradigms (*n* = 2), affective/emotional-processing paradigms (*n* = 3), and other tasks (*n* = 1) were less common. Among oddball/deviance articles, 17 used ERP analyses only and 3 combined ERP and qEEG approaches. Attention/executive control articles used ERP analyses only (*n* = 14), qEEG only (*n* = 5), or both approaches (*n* = 2).

**Figure 4:**
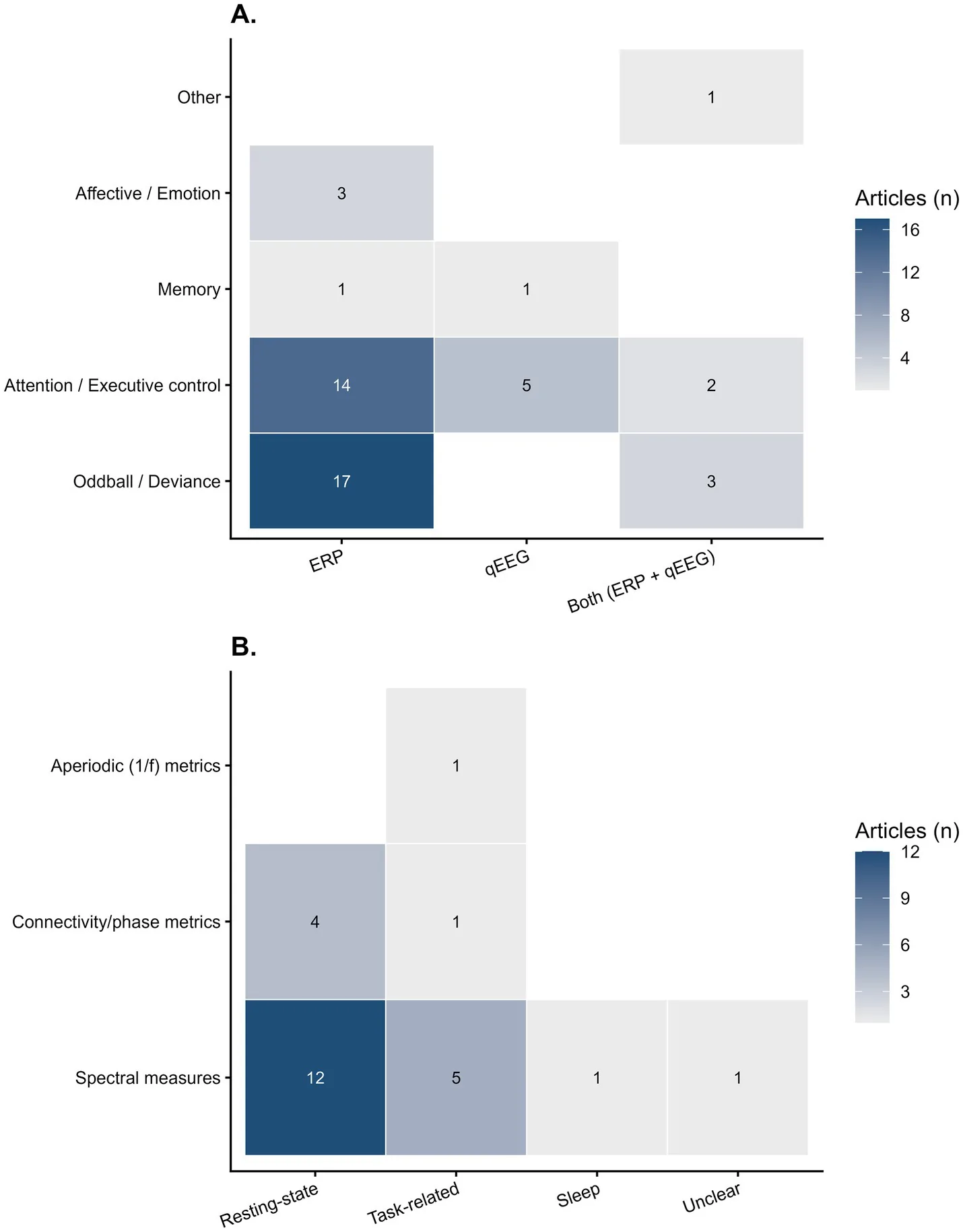
EEG paradigms and quantitative EEG (qEEG) analytic families across included articles. **(A)** Task-based EEG paradigms grouped by paradigm class and EEG approach: Event-related potentials (ERP), qEEG, or both. **(B)** Primary qEEG analytic family by recording context. Each qEEG article was assigned one primary analytic family and context based on its principal qEEG analysis. Cell values indicate the number of articles in each category.

#### 3.3.2 Quantitative EEG measures and recording context

For Figure 4B, each of the 25 articles reporting qEEG measures was assigned to one primary qEEG analysis family and recording context. Spectral power was the most frequently reported analysis family in 19 articles, connectivity or phase-based measures in 5, and aperiodic (1/f) measures in 1. The corresponding qEEG measures were derived primarily from resting-state recordings in 16 articles, task-related recordings in 7, sleep EEG in 1, and an unclear recording context in 1.

### 3.4 Cognitive and related outcomes

Cognitive and related outcomes were grouped into three non-mutually exclusive categories. Standardized cognitive tests were reported in 39 articles: 21 using ERP only, 15 using qEEG only, and 3 using both approaches. Subjective measures were reported in 27 articles: 14 using ERP only, 11 using qEEG only, and 2 using both. Behavioral performance during experimental tasks was reported in 30 articles: 22 using ERP only, 5 using qEEG only, and 3 using both approaches (Figure 5).

**Figure 5:**
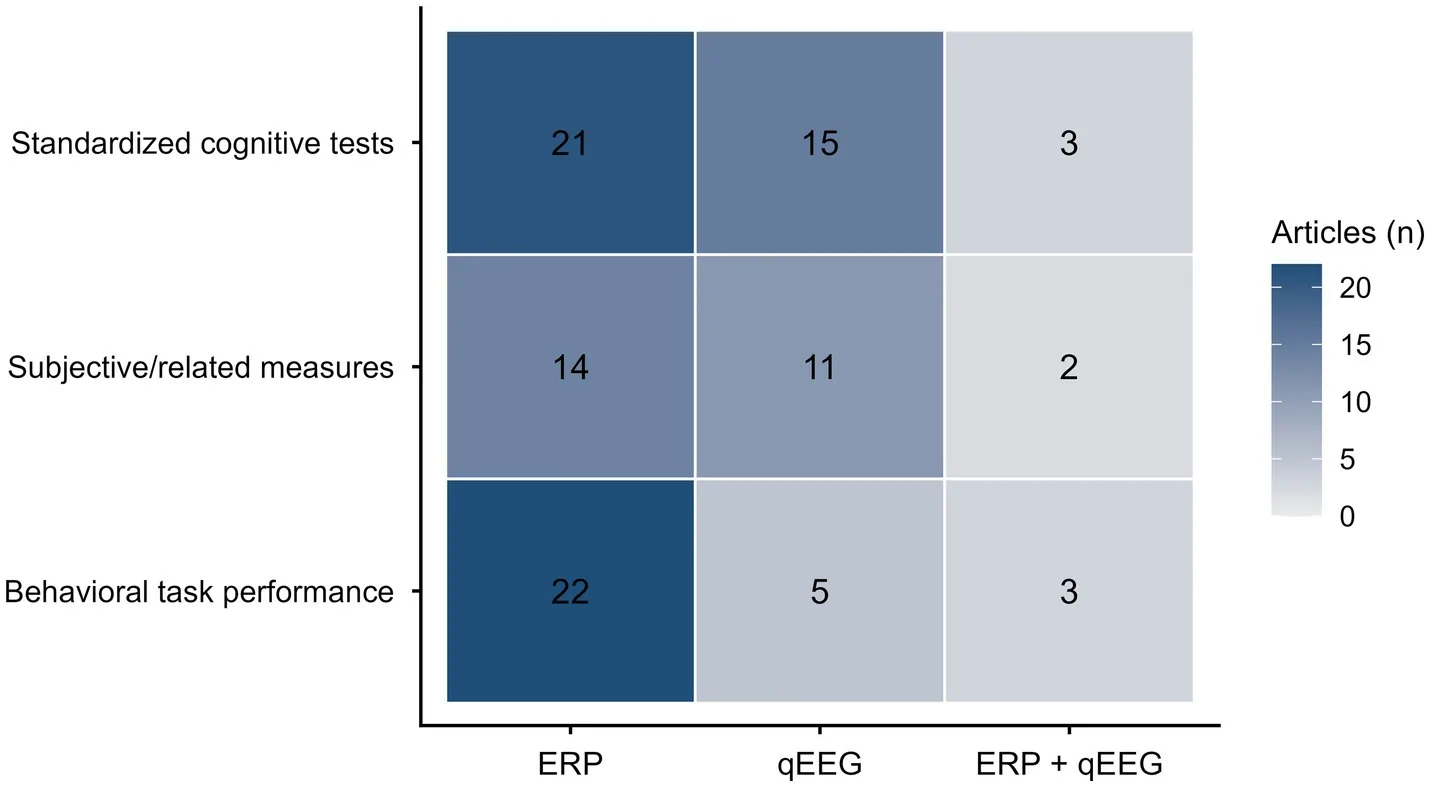
Cognitive and related outcome measures by EEG approach. The heatmap shows the number of articles reporting standardized cognitive tests, subjective or related patient-reported measures, or behavioral performance during experimental tasks, stratified by EEG approach: event-related potentials (ERP), quantitative EEG (qEEG), or both. Outcome categories were not mutually exclusive, and articles could contribute to more than one row. Three articles did not contribute to the plotted categories: one used a clinician-rated cognitive classification, and two did not report a separate cognitive outcome. Cell values indicate article counts.

### 3.5 Narrative synthesis of EEG findings and EEG-cognition relationships

Cross-study findings are summarized in six non-mutually exclusive EEG categories and stratified by cancer context in Table 3. Mixed samples are identified separately where relevant. Article counts do not represent independent cohorts because several articles reported distinct analyses of known or probably overlapping samples, including the Anderson et al. (2018, 2019, 2020), Lähteenmäki (1999), Lähteenmäki et al. (2001), Kreukels et al. (2005, 2006, 2008a, 2008b), Prashad et al. (2023), Melara et al. (2023, 2025), and Wolthuis et al. (2021, 2022) reports.

**Table 3:** EEG evidence on cognition, stratified by cancer context.

EEG approach	Cancer context and evidence base	Cross-study EEG pattern	EEG-cognition evidence	Interpretation and remaining uncertainty
P300-focused and oddball/deviance ERPs	CNS/intracranial tumors: 2 articles; mixed primary/metastatic intracranial tumors: 1 articleᵃ	Findings ranged from stable P300 measures during treatment to prolonged N2/P3 latency in cognitively impaired patients and increased P3b amplitude after an intervention.	Prolonged N2/P3 latency corresponded with clinician-rated impairment in one article; direct associations were either not tested or not supported in the other two.	The small, heterogeneous evidence base does not establish a consistent ERP pattern or its cognitive relevance.
	Non-CNS cancers 24 articles^b^	Longer P300 latency and lower amplitude were reported most often; shorter latency and null findings also occurred across populations and assessment times.	ERP measures were associated with cognitive test or task performance in some articles, but findings varied and direct associations were often not tested.	Longer P300 latency emerged as the most recurrent finding, but differences in paradigms, treatment stages, and outcomes limit its interpretation as a cognitive marker.
Other task-evoked ERPs	CNS/intracranial tumors: 2 articles^c^	Task-specific findings concerned response selection and motor preparation in one article and visual character processing in the other; the paradigms did not overlap.	Neither article directly related ERP measures to independently assessed cognitive outcomes.	Two small, task-specific articles do not support a cross-study ERP pattern.
	Non-CNS cancers: 11 articles; mixed tumor/TBI sample: 1 articleᵈ	Differences were reported in early sensory, attention, working-memory, response-inhibition, error-monitoring, and emotional-processing ERPs, but specific components were rarely replicated.	Selected measures were related to task performance, cognitive complaints, or cognitive test outcomes, although ERP differences were also reported with preserved behavioral performance.	ERPs may be sensitive to task-related processing differences, but no component-outcome pattern emerged.
Resting-state and sleep spectral EEG	CNS/intracranial tumors: 2 articles^e^	Both articles reported increased low-frequency activity, suggesting preliminary convergence on spectral slowing across waking and sleep recordings.	Greater theta activity was related to poorer language performance in one glioma cohort; the other article reported parallel EEG and cognitive severity differences without direct association testing.	Spectral slowing is a tentative shared finding, but its cognitive relevance was only tested directly in one cohort.
	Non-CNS cancers: 10 articles^f^	Alpha-related measures were examined most often, but their direction varied; findings in other frequency bands were isolated or treatment-specific.	Selected spectral measures were related to cognitive test performance or cognitive complaints, but associations varied across frequency bands and scalp regions.	No reproducible spectral-cognitive pattern emerged, and cognitive associations could not be clearly separated from symptom-related or intervention-related changes.
Task-related spectral and aperiodic EEG	CNS/intracranial tumors: 1 article^g^	Broader task-evoked source activation was reported during visual character processing.	No direct relation to reported cognitive difficulties was tested; some task-related EEG measures varied with tumor volume or grade.	A single article is insufficient to establish cognitive relevance.
	Non-CNS cancers: 5 articles; mixed samples: 2 articlesʰ	Measures included task-related alpha and broadband power, workload classification, and aperiodic slope; tasks and findings did not converge.	Less negative aperiodic slope, interpreted as greater neural noise, was related to poorer executive control and alerting in one longitudinal cohort. Other findings primarily concerned task demand, workload, or fatigue.	The aperiodic finding is a specific candidate, but it has not been independently replicated.
Connectivity and network measures	CNS/intracranial tumors: 4 articles^i^	Altered coherence, phase synchrony, and network organization were reported, but patterns varied by lesion location, task, frequency band, and assessment time.	Language showed the clearest preliminary relationship with network measures; evidence for executive outcomes was weaker.	Theta-band network organization in relation to language was the clearest candidate, but this evidence rests mainly on one longitudinal glioma cohort.
	Non-CNS cancers: 1 article^j^	Lower frontocentroparietal coherence in two breast cancer chemotherapy groups than in controls; differences between treatment regimens were inconclusive.	Cognitive and connectivity findings were reported in parallel without direct association testing.	Cognitive relevance and reproducibility remain uncertain.
Quantified background EEG in acute neurotoxicity	Non-CNS cancers: 2 articles^k^	Both articles reported treatment-related background slowing: reduced alpha and increased delta after interleukin-2 treatment, and parieto-occipital slowing that sometimes preceded methotrexate-associated encephalopathy.	Cognitive or clinical changes were reported alongside EEG changes, but neither article directly related EEG measures to cognitive test scores.	Quantified background slowing may be sensitive to acute neurotoxicity, but evidence that it predicts later cognitive outcomes is lacking.

CNS, central nervous system; EEG, electroencephalography; ERP, event-related potential; TBI, traumatic brain injury. Counts indicate article contributions rather than independent cohorts. Categories are non-mutually exclusive, so counts are not additive and do not need to match the primary classifications in Figure 4. Mixed contexts are identified within the relevant rows. Detailed study-level findings and references are provided in Supplementary material 3.

Articles contributing to each row. ^a^Bian et al. (2019), Olbrich et al. (1986), Schellart et al. (2011). ^b^Brace et al. (2019), Caraceni et al. (1993), Heukrodt et al. (1988), Hung et al. (2020), Järvelä et al. (2011), Kam et al. (2016), Krawutschke et al. (2025), Kreukels et al. (2005), (2006), (2008a), (2008b), Kroczka et al. (2022), Lähteenmäki et al. (2001), Lin et al. (2022), Moore et al. (1992), Münte et al. (2004), Pace et al. (1994), Parageorgiou et al. (2000), Pietrangeli et al. (1990), Schagen et al. (2001), Siddiqui et al. (1992), Überall et al. (1996), Xu et al. (2020), Zhang et al. (2019). ^c^Quik et al. (2012), Yang et al. (2022). ^d^Anderson et al. (2018), (2019), (2020), Berger et al. (2023), Gan et al. (2019), Li et al. (2022), Melara et al. (2023), Schoenberg et al. (2024), Simó et al. (2018), Swainston et al. (2021), Wirkner et al. (2017), mixed sample: Solbakk et al. (2014). ^e^Arapova et al. (2022), Wolthuis et al. (2022). ^f^Cao et al. (2017), Clavio et al. (2004), Ercoli et al. (2013), Ercoli et al. (2015), Hunter et al. (2014), Kam et al. (2016), Schagen et al. (2001), Sideroff et al. (2023), van Dyk et al. (2017), Vasaghi Gharamaleki et al. (2022). ^g^Yang et al. (2022). ^h^Lähteenmäki (1999), Melara et al. (2025), Moore et al. (2014), Prashad et al. (2023), Vasaghi Gharamaleki et al. (2022), mixed samples: Mathan et al. (2010), Solbakk et al. (2014). ^i^Kuleva et al. (2022), Sadaghiani et al. (2019), Wolthuis et al. (2021), Yang et al. (2022). ^j^Vasaghi Gharamaleki et al. (2022). ^k^Ch’ien et al. (1981), Pace et al. (1994).

The evidence differed by cancer context. Studies of primary CNS and other intracranial tumors primarily examined EEG variation in relation to tumor-related factors and domain-specific functioning, particularly language and executive performance (Sadaghiani et al., 2019; Wolthuis et al., 2021, 2022; Yang et al., 2022). Studies of non-CNS cancers more often examined treatment-related or longitudinal changes in ERPs and spectral activity, particularly in breast cancer, hematologic malignancies, and childhood cancer (Heukrodt et al., 1988; Kam et al., 2016; Kreukels et al., 2005, 2006, 2008a, 2008b; Järvelä et al., 2011; Melara et al., 2023, 2025). No single EEG pattern emerged across evidence categories. The presence and direction of findings varied considerably across EEG approaches, study populations, treatment stages, and assessment times.

Overall, 21 of 60 articles (35.0%) reported at least one direct EEG–outcome association that was statistically significant according to the threshold used in the original article. Four additional articles reported qualified or mixed associations, two tested direct associations without finding a statistically significant relationship, and 33 did not test a direct association. Outcomes included standardized cognitive-test performance, subjective cognition or related symptoms, clinician-rated cognitive classification, and behavioral task performance. Evidence based specifically on standardized cognitive tests administered independently of the EEG task was more limited. Many articles instead reported task-related EEG modulation, group differences, or parallel longitudinal changes without directly testing whether individual differences in EEG measures were associated with cognitive outcomes (e.g., Caraceni et al., 1993; Bian et al., 2019; Lin et al., 2022; Sideroff et al., 2023). Thus, direct EEG–cognition associations were examined less consistently than group-, task-, treatment-, or time-related EEG variation.

## 4 Discussion

This scoping review identified 60 articles using ERP and/or qEEG to examine cognitive functioning in people with cancer. Most articles used task-based ERPs, particularly oddball and attention or executive-control paradigms, whereas resting-state, connectivity, and other qEEG approaches were less common. Publication output increased markedly after 2015, but direct associations between EEG measures and cognitive outcomes were tested in fewer than half of the articles.

The evidence was largely exploratory. Fifty-three of the 60 articles (88.3%) included fewer than 50 cancer participants, and more than half used cross-sectional designs. EEG acquisition, preprocessing, paradigms, analytic measures, and cognitive outcomes varied considerably, while several articles examined multiple EEG features, electrodes, time windows, or cognitive endpoints. Interpretation was also complicated by potential confounding from demographic, clinical, treatment-related, and symptom factors that differed across studies and were not always incorporated into the analyses. These features limit confidence in cross-study patterns and causal interpretations. The findings should therefore be viewed as hypothesis-generating rather than as evidence for established EEG markers of CRCI.

The size of previous CRCI reviews has varied widely according to the populations, treatments, cognitive outcomes, and study designs considered (Bray et al., 2018; Cerulla Torrente et al., 2020; Dijkshoorn et al., 2021; Saita et al., 2023). Neuroimaging reviews have also described a methodologically heterogeneous literature, particularly in non-CNS cancer and breast cancer populations (de Ruiter and Schagen, 2013; Amidi and Wu, 2019; Sousa et al., 2020; Niu et al., 2021; Chen et al., 2025). Direct numerical comparisons are limited by differences in review scope and eligibility criteria.

Cancer populations were unevenly represented. Breast cancer was the most frequently studied diagnosis, followed by hematologic malignancies and brain tumors, while other solid tumors were represented in relatively few articles. A similar concentration in breast cancer has been reported in structural neuroimaging, including one review in which 77% of studies of adult non-CNS cancers involved breast cancer populations (Amidi and Wu, 2019). Only nine articles included children or adolescents, and five of these included mixed pediatric and adult samples. The limited evidence restricts conclusions about CRCI in younger populations. More broadly, the concentration of research in a small number of cancer populations limits generalizability across diagnoses, age groups, and treatment contexts.

Studies of primary CNS and other intracranial tumors addressed different clinical and mechanistic questions from studies of non-CNS cancers. In CNS and intracranial tumors, EEG findings were more often examined in relation to tumor-related neurological factors. Reported findings included focal or diffuse slowing, altered phase synchrony, and differences in network organization examined in relation to tumor location, neurological status, or language and executive outcomes (Wolthuis et al., 2021, 2022; Arapova et al., 2022; Kuleva et al., 2022; Yang et al., 2022). However, the contributions of tumor burden, edema, surgery, seizures, antiepileptic medication, radiotherapy, and adjuvant treatment could not always be separated. In non-CNS cancers, the focus was more often on treatment-related neural change. Several articles lacked a pretreatment baseline or an appropriate cancer comparison group, and consideration of fatigue, mood, endocrine treatment, and practice effects varied.

P300 findings varied across articles. Longer P300 latency and/or lower amplitude were reported in several childhood cancer, breast cancer, radiotherapy, and acute treatment studies (Heukrodt et al., 1988; Pietrangeli et al., 1990; Caraceni et al., 1993; Pace et al., 1994; Lähteenmäki et al., 2001; Kreukels et al., 2006, 2008a; Kam et al., 2016; Xu et al., 2020; Kroczka et al., 2022), while other studies reported stable P300, null group differences, or shorter latency (Parageorgiou et al., 2000; Schagen et al., 2001; Kreukels et al., 2005; Bian et al., 2019; Brace et al., 2019). P300 latency was associated with global cognitive classification, IQ, or academic outcomes in some articles (Olbrich et al., 1986; Heukrodt et al., 1988; Kreukels et al., 2008b), but these findings were not consistent across populations or assessment points. Differences in stimulus modality, target probability, electrode selection, peak definition, age, and treatment timing further limit cross-study comparison.

Other task-related ERP articles reported differences during tasks involving early sensory processing, attentional selection, working memory filtering, inhibition, error monitoring, and emotional processing (Simó et al., 2018; Anderson et al., 2019, 2020; Gan et al., 2019; Swainston et al., 2021; Li et al., 2022; Yang et al., 2022; Melara et al., 2023). In several articles, ERP differences occurred without corresponding differences in behavioral performance (Solbakk et al., 2014; Simó et al., 2018; Anderson et al., 2019; Swainston et al., 2021). One possible interpretation is that these findings reflect compensation or subclinical vulnerability, but limited statistical power, ceiling effects, and multiple testing provide alternative explanations. Studies with calibrated task difficulty and prospective functional outcomes would help distinguish these explanations.

Resting and task-related qEEG findings were diverse and rarely replicated across independent cohorts. In two reports from one glioma cohort, focal theta and theta-band network organization were related to language outcomes (Wolthuis et al., 2021, 2022), while high-alpha phase-synchrony modulation showed a qualified relationship with set-shifting performance in another study (Sadaghiani et al., 2019). In non-CNS cancer, direct spectral associations were heterogeneous and included standardized cognitive performance, subjective cognition, fatigue, and mood, often using exploratory combinations of frequency band and scalp region (Ercoli et al., 2013; Hunter et al., 2014; Moore et al., 2014; Cao et al., 2017; van Dyk et al., 2017). In a subsequent intervention study, change in delta power was not associated with improvement in cognitive complaints, whereas an increased anterior–posterior alpha gradient was associated with later improvement in self-reported cognition (Ercoli et al., 2015). In one longitudinal cohort, aperiodic slopes became flatter from before to after treatment in patients relative to controls, which the authors interpreted as increased neural noise. Flatter slopes were also associated with poorer executive control and alerting performance (Melara et al., 2025).

Fatigue was among the outcomes associated with qEEG, but these associations should not be interpreted as cognition specific. A systematic review of 10 MRI studies in breast cancer found associations between fatigue and white-matter and functional changes, particularly in frontoparietal and attention-related networks, sometimes without reduced task performance (Arya et al., 2021). These findings suggest that fatigue and cognitive functioning may involve partly overlapping neural systems, although the relevance of these fMRI findings to EEG remains uncertain.

A central finding is the gap between detecting an EEG abnormality and validating an EEG marker of cognition. Overall, 21 of 60 articles (35%) reported at least one direct EEG-outcome association that met the statistical significance threshold used in the original article. Outcomes included performance on standardized cognitive tests, subjective cognition or related symptoms, clinician-rated cognitive classification, and behavioral task performance. Of these, associations involving standardized cognitive tests administered independently of the EEG task were less common. Many articles instead reported task-related EEG modulation, group differences, or parallel longitudinal changes without testing whether individual EEG values tracked cognitive outcomes or cognitive change (Caraceni et al., 1993; Bian et al., 2019; Lin et al., 2022; Sideroff et al., 2023). These findings do not establish a replicated or clinically interpretable EEG marker of CRCI.

### 4.1 Future directions

Several directions for future research may strengthen this field. Larger and longitudinal designs are needed to clarify the temporal relationship between neural and cognitive changes. Most existing studies are cross-sectional and cannot distinguish pre-existing differences from treatment-related effects or track recovery trajectories across survivorship.

The potential of EEG for risk stratification and prediction remains largely unexplored. Most studies report group-level differences rather than attempting individual-level prediction or prognosis. Future research could evaluate whether EEG features predict clinically meaningful outcomes such as incident cognitive impairment or persistence of symptoms, ideally using independent validation samples.

Greater methodological convergence may improve comparability across studies. Future studies should prespecify primary EEG features and cognitive outcomes, report acquisition and preprocessing procedures transparently, correct appropriately for multiple testing, and account for key clinical and symptom-related confounders. Although CRCI-specific EEG standards do not yet exist, related fields provide useful examples, including resting-state EEG recommendations for clinical and cognitive-impairment research (Babiloni et al., 2020, 2021) and reproducible ERP resources such as ERP CORE (Kappenman et al., 2021).

### 4.2 Limitations

This review has several limitations. Categorizing EEG paradigms, qEEG analyses, and cognitive outcomes necessarily simplified heterogeneous methods, and some articles used mixed approaches that could not be represented fully by single labels. The review was restricted to English-language, peer-reviewed publications identified through three databases and reference-list screening; relevant non-English or unpublished evidence may therefore have been missed. In line with the mapping aim of this scoping review, we did not conduct a formal risk-of-bias assessment and therefore do not provide article-level judgments or an overall certainty rating. Finally, although the protocol was registered as a systematic review, the mapped heterogeneity identified during screening and data charting supported reporting the work as a scoping review in accordance with PRISMA-ScR.

## 5 Conclusion

EEG studies of CRCI that include cognitive outcomes increased over time, but samples have generally been small and methods heterogeneous. Most articles used task-based ERPs, particularly oddball and attention or executive control paradigms. Resting-state and other qEEG approaches were less common, and connectivity or microstate analyses remained limited. Cognitive functioning was assessed using standardized cognitive tests, self-reported measures, and behavioral task performance. More consistent methods, larger longitudinal studies, and closer integration of EEG and cognitive outcomes are needed to support cumulative evidence. EEG may help clarify the neural processes associated with CRCI, but the current evidence is insufficient to support biomarker or risk-stratification applications.

## Data Availability

The original contributions presented in the study are included in the article/Supplementary material. Further inquiries can be directed to the corresponding author.
