# Supplementary material for: Functional brain activity in cancer-related cognitive impairment: a scoping review of EEG studies
Source: Front Hum Neurosci. 2026 Oct 15;20:1851560. doi: 10.3389/fnhum.2026.1851560 (PMC13716737; doi:10.3389/fnhum.2026.1851560)
Supplement: Supplementary file 1 [file Data_Sheet_1.pdf]

MEDLINE
Platform: Ovid
Coverage: From database inception
Initial search date: 26 July 2023
Update search date: 11 February 2025

1. exp Neoplasms/ OR
exp Medical Oncology/ OR
Psycho-Oncology/ OR
cancer*.ti,ab,kw. OR
leuk?emia.ti,ab,kw. OR
malign*.ti,ab,kw. OR
oncolog*.ti,ab,kw. OR
psycho-oncology.ti,ab,kw. OR
psychooncology.ti,ab,kw. OR
tumor.ti,ab,kw. OR
tumour.ti,ab,kw. OR
chemo*.ti,ab,kw.

2. exp Electroencephalography/ OR
Event-Related Potentials, P300/ OR
Evoked Potentials/ OR
electroencephalogra*.ti,ab,kw. OR
Event Related Potentials.ti,ab,kw. OR
Evoked Potential*.ti,ab,kw. OR
EEG.ti,ab,kw. OR
qEEG.ti,ab,kw.

3. Cognition/ OR
Neuropsychology/ OR
Chemotherapy-Related Cognitive Impairment/ OR
cognit*.ti,ab,kw. OR
"chemo brain".ti,ab,kw. OR
neuropsycholog*.ti,ab,kw.

4. 1 AND 2 AND 3

PsycINFO
Platform: APA PsycNet
Coverage: From database inception
Initial search date: 26 July 2023
Update search date: 11 February 2025

1 Any Field: neoplasms OR
Any Field: Chemotherapy-Related Cognitive Impairment OR
Any Field: Medical Oncology OR
Any Field: Psycho-Oncology OR
Any Field: cancer OR
Any Field: leukemia OR
Any Field: malign* OR
Any Field: oncolog* OR
Any Field: psychooncology OR
Any Field: tumor OR
Any Field: tumour OR
Any Field: chemo*

2 Any Field: Electroencephalography OR
Any Field: "Event-Related Potentials" OR
Any Field: P300 OR
Any Field: "Brain wave*" OR
Any Field: electroencephalogra* OR
Any Field: "Event Related Potentials" OR
Any Field: eeg OR
Any Field: "evoked potentials"

3 Any Field: cognition OR
Any Field: Neuropsychology OR
Any Field: cognit* OR
Any Field: "chemo brain" OR
Any Field: "chemo fog" OR
Any Field: neuropsycholog* OR
Any Field: "cancer related cognitive impairment"

Searches 1–3 were combined using APA PsycNet's "Combine Searches" function with AND.

Web of Science Core Collection
Platform: Clarivate
Indexes included: Science Citation Index Expanded, Social Sciences Citation Index, Arts & Humanities Citation Index, Emerging Sources Citation Index, Conference Proceedings Citation Index–Science, Conference Proceedings Citation Index–Social Sciences & Humanities, Book Citation Index–Science, Book Citation Index–Social Sciences & Humanities, Current Chemical Reactions, and Index Chemicus.
Coverage: From database inception
Initial search date: 26 July 2023
Update search date: 11 February 2025

#1 ALL=(Neoplasm* OR
“Medical Oncology” OR
cancer* OR
leukemia OR
malign* OR
oncolog* OR
“psycho-oncology” OR
psychooncology OR
tumor* OR
tumour* OR
chemo*)

#2 ALL=(Electroencephalography OR
“Event-Related Potentials” OR
“P300” OR
“Evoked Potential*” OR
electroencephalogra* OR
“Event Related Potentials” OR
EEG* OR
qEEG)

#3 ALL=(“Chemotherapy-Related Cognitive Impairment” OR
Cognition OR
Neuropsychology OR
cognit* OR
"chemo brain" OR
neuropsycholog*)

#4 #1 AND #2 AND #3
