# Supplementary material for: Functional brain activity in cancer-related cognitive impairment: a scoping review of EEG studies
Source: Front Hum Neurosci. 2026 Oct 15;20:1851560. doi: 10.3389/fnhum.2026.1851560 (PMC13716737; doi:10.3389/fnhum.2026.1851560)
Supplement: Supplementary file 3 [file Table_2.pdf]

Supplementary Material 3. Characteristics and findings of included articles

Panel A. Study and sample characteristics

Citation	Population	Country	Design and assessment timing	Sample and comparison
Anderson et al. (2018)	Non-CNS; Hematologic malignancies (myelodysplastic syndrome, non-Hodgkin lymphoma, multiple myeloma); Adults	USA	Cross-sectional; During chemotherapy (n = 6) or best supportive care (n = 8)	Patients: 14; Comparators: 13; Patients vs healthy controls
Anderson et al. (2019)	Non-CNS; Hematologic malignancies (NHL, MDS, AML, CLL, ALL, MM); Adults	USA	Cross-sectional; Pre-treatment	Patients: 28; Comparators: 15; Patients vs healthy controls
Anderson et al. (2020)	Non-CNS; Hematologic malignancies (mainly NHL, AML, MDS, CLL, ALL, MM); Adults	USA	Longitudinal, prospective (pilot); Mixed: pre-treatment baseline and during treatment follow-up	Patients: 18; Comparators: 12; Patient treatment groups vs healthy controls; Longitudinal
Arapova et al. (2022)	Primary CNS/intracranial; Brain tumor (frontal lobe glioma/tumors); Adults	Russia	Cross-sectional; Pre-surgery	Patients: 20; Comparators: 10; Patient severity groups vs healthy controls
Berger et al. (2023)	Non-CNS; Gastrointestinal cancer (colorectal cancer); Adults	USA	Longitudinal, prospective; Mixed: pre-chemotherapy baseline and during treatment follow-up	Patients: 24; Comparators: 14; Patient treatment groups vs healthy controls; Longitudinal
Bian et al. (2019)	Primary CNS/intracranial; Brain tumor (high-grade glioma (WHO III/IV)); Adults	China	Longitudinal, prospective; Mixed: pre-treatment baseline, during treatment, and post-treatment follow-up	Patients: 18; Within-patient longitudinal (pre-post)
Brace et al. (2019)	Non-CNS; Childhood cancer (ALL); Children	USA	Cross-sectional; Early survivorship (<1 year)	Patients: 8; Comparators: 8; Patients vs healthy controls
Cao et al. (2017)	Non-CNS; Gastrointestinal cancer (Esophageal cancer with depression); Adults	China	Cross-sectional; No chemotherapy; timing relative to other cancer treatment not reported	Patients: 65; Comparators: 62; Patients vs healthy controls and patient subgroup comparison
Caraceni et al. (1993)	Non-CNS; Melanoma (metastatic melanoma); Adults	Italy	Longitudinal, prospective; During treatment and immediate post-treatment	Patients: 7; Within-patient longitudinal (pre-post)

Panel A. Study and sample characteristics (continued)

Citation	Population	Country	Design and assessment timing	Sample and comparison
Ch'ien et al. (1981)	Non-CNS; Hematologic malignancies (ALL); Children	USA	Prospective treatment cohort with nested longitudinal case series; During treatment and longitudinal follow-up	Patients: 11; Nested case series within prospective treatment cohort
Clavio et al. (2004)	Non-CNS; Hematologic malignancy (low-risk myelodysplastic syndromes); Adults	Italy	Prospective, open-label, single-arm intervention; During treatment	Patients: 8; Within-patient longitudinal (pre-post)
Ercoli et al. (2013)	Non-CNS; Breast cancer (stage 0-III survivors); Adults	USA	Prospective single-arm feasibility intervention (cognitive rehabilitation); Survivorship (≥1 year)	Patients: 8; Within-patient longitudinal (pre-post)
Ercoli et al. (2015)	Non-CNS; Breast cancer (stage 0-III survivors); Adults	USA	Randomized controlled trial (2:1 cognitive rehabilitation vs wait list); Survivorship (≥1 year)	Patients: 29; Patient intervention vs patient wait-list control; Longitudinal
Gan et al. (2019)	Non-CNS; Breast cancer (stage II-IV); Adults; Age not reported	China	Prospective within-patient pre-post study; Pre-treatment and post-treatment	Patients: 17; Within-patient longitudinal (pre-post)
Heukrodt et al. (1988)	Non-CNS; Childhood non-CNS cancers (ALL and extracranial solid tumors); Mixed/unclear age; Age at testing not reported	USA	Cross-sectional retrospective survivor study; Survivorship (≥1 year)	Patients: 42; Comparators: 14; Patient diagnostic groups vs healthy control; Cross-sectional
Hung et al. (2020)	Non-CNS; Colon cancer (stage II–III); Adults	Canada	Longitudinal, prospective pilot; Mixed: pre/early-treatment baseline, end of treatment, and early survivorship follow-up	Patients: 10; Within-patient longitudinal (three timepoints, n=6)
Hunter et al. (2014)	Non-CNS; Breast cancer; Adults	USA	Cross-sectional, exploratory pilot; Survivorship (mean >2 years since chemotherapy)	Patients: 27; Single patient group

Panel A. Study and sample characteristics (continued)

Citation	Population	Country	Design and assessment timing	Sample and comparison
Järvelä et al. (2011)	Non-CNS; Childhood non-CNS cancers (ALL, NHL, Wilms tumor, germ-cell tumor, osteosarcoma); Mixed pediatric/adult; predominantly pediatric	Finland	Longitudinal, prospective matched-control study; Mixed: diagnosis/early treatment and post-treatment follow-up	Patients: 25; Comparators: 21; Patients vs matched healthy controls; Longitudinal
Kam et al. (2016)	Non-CNS; Breast cancer (stage II–III, postmenopausal survivors); Adults	Canada	Cross-sectional matched-control study; Mixed post-treatment survivorship (3 months–3 years)	Patients: 18; Comparators: 12; Patients vs healthy controls
Krawutschke et al. (2025)	Non-CNS; Mixed cancer types (breast, melanoma, lung, etc.); Adults	Germany	Single-arm longitudinal intervention substudy with waiting-period assessment; Mixed: active treatment and survivorship/remission	Patients: 18; Within-patient waiting-period and pre-post intervention
Kreukels et al. (2005)[a]	Non-CNS; Breast cancer; Adults	Netherlands	Cross-sectional; Survivorship (3–6 years post-treatment)	Patients: 25; Comparators: 23; Chemotherapy-treated patients vs cancer controls
Kreukels et al. (2006)[a]	Non-CNS; Breast cancer; Adults	Netherlands	Cross-sectional; Survivorship (approximately 3–5 years post-treatment)	Patients: 28; Comparators: 23; Chemotherapy regimen groups vs cancer controls
Kreukels et al. (2008a)[a]	Non-CNS; Breast cancer; Adults	Netherlands	Cross-sectional; Survivorship (3–6 years post-treatment)	Patients: 53; Comparators: 23; Chemotherapy regimen groups vs cancer controls
Kreukels et al. (2008b)[a]	Non-CNS; Breast cancer; Adults	Netherlands	Longitudinal follow-up of a cross-sectional cohort; Survivorship (T1 6–12 months; T2 approximately 5 years post-treatment)	Patients: 26; Within-patient subgroup comparison based on earlier cognitive status
Kroczka et al. (2022)	Non-CNS; Childhood acute lymphoblastic leukemia (ALL); Mixed pediatric/adult	Poland	Cross-sectional; Survivorship (1.5–21.8 years post-treatment)	Patients: 136; Comparators: 58; Patients vs mixed controls; Protocol and radiotherapy subgroup comparisons

Panel A. Study and sample characteristics (continued)

Citation	Population	Country	Design and assessment timing	Sample and comparison
Kuleva et al. (2022)	Primary CNS/intracranial; CNS tumor (cavernous sinus meningioma compressing the mediobasal temporal lobe); Adults	Russia	Cross-sectional multimodal study; Treatment status not reported	Patients: 14; Comparators: 9; Patient subgroups vs healthy controls
Li et al. (2022)	Non-CNS; Breast cancer; Adults	China	Prospective within-patient pre–post study; Pre-treatment baseline and post-treatment follow-up	Patients: 22; Within-patient longitudinal (pre–post)
Lin et al. (2022)	Non-CNS; Colorectal cancer; Adults	China	Randomized controlled trial (per-protocol analysis); Post-treatment; interval not reported	Patients: 55; Patient intervention vs patient controls; Longitudinal randomized trial
Lähteenmäki (1999)[b]	Non-CNS; Childhood leukemia/lymphoma and extracranial solid tumors; Adolescents	Finland	Cross-sectional case-control study; Survivorship (≥1 year)	Patients: 19; Comparators: 10; Patient subgroups vs healthy controls
Lähteenmäki et al. (2001)[b]	Non-CNS; Childhood leukemia/lymphoma and extracranial solid tumors; Adolescents	Finland	Cross-sectional case-control pilot study; Survivorship (≥1 year)	Patients: 19; Comparators: 10; Patient subgroups vs healthy controls; Ancillary sibling academic comparison
Mathan et al. (2010)	Mixed CNS and non-CNS; Mixed: malignant brain tumor (n=5; stages I–III) and breast cancer (n=5; stages I–III); Adults	USA	Cross-sectional proof-of-concept study; Survivorship (≥1 year)	Patients: 10; Within-participant task-condition comparison
Melara et al. (2023)[c]	Non-CNS; Breast cancer (stage 0–IIA); Adults	USA	Prospective longitudinal patient–control study; Pre-treatment baseline and post-treatment follow-up	Patients: 15; Comparators: 24; Patients vs healthy controls; Longitudinal
Melara et al. (2025)[c]	Non-CNS; Breast cancer (stage 0–IIA); Adults	USA	Prospective longitudinal patient–control study; Pre-treatment baseline and post-treatment follow-up	Patients: 20 (paired-click); 19 (ANT-R); Comparators: 32 (paired-click); 29 (ANT-R); Patients vs healthy controls; Longitudinal

Panel A. Study and sample characteristics (continued)

Citation	Population	Country	Design and assessment timing	Sample and comparison
Moore et al. (1992)	Non-CNS; Mixed childhood cancers, predominantly leukemia; brain tumor cases excluded; Mixed pediatric/adult	USA	Cross-sectional treatment-group comparison; Survivorship (≥5 years off therapy)	Patients: 27; Patient treatment groups (CNS-treatment intensity); Cross-sectional
Moore et al. (2014)	Non-CNS; Breast cancer (stage I–III); Adults	USA	Prospective longitudinal pilot patient–control study; Mixed: pre-treatment baseline, during treatment, and post-treatment follow-up	Patients: 8; Comparators: 8; Longitudinal patients vs matched healthy controls
Münte et al. (2004)	Non-CNS; Well-differentiated thyroid carcinoma; Adults	Germany	Prospective within-patient thyroid-state comparison (fixed order); During treatment-related thyroid hormone withdrawal and short-term recovery	Patients: 15; Within-patient longitudinal thyroid-state comparison
Olbrich et al. (1986)	Primary CNS/intracranial; Mixed primary and supratentorial tumors, including gliomas, meningiomas, metastases, and other tumors; Adults	Germany	Cross-sectional clinical group comparison; Pre-treatment (before intracranial surgery, CNS irradiation, or cytostatic treatment)	Patients: 21; Comparators: 12; Patient cognitive-severity groups vs age-matched hospital controls
Pace et al. (1994)	Non-CNS; Mixed non-CNS solid tumors: melanoma (n=13), renal cancer (n=5), and colorectal cancer (n=2); Adults	Italy	Prospective uncontrolled pre–post study with healthy baseline reference; During treatment and immediate post-treatment	Patients: 18 analyzed (20 enrolled); Comparators: 15; Within-patient longitudinal pre–post comparison with healthy baseline reference
Parageorgiou et al. (2000)	Non-CNS; Small-cell lung cancer without brain metastases; Adults	Greece	Prospective uncontrolled pre–post study; Post-systemic treatment; Pre- and short-term post-cranial irradiation	Patients: 11; Within-patient longitudinal (pre–post)

Panel A. Study and sample characteristics (continued)

Citation	Population	Country	Design and assessment timing	Sample and comparison
Pietrangeli et al. (1990)	Non-CNS with/without CNS metastases; Lung cancer; brain metastases present in 5/9 patients; Adults	Italy	Prospective uncontrolled pre–post study; Pre- and short-term post-cranial irradiation	Patients: 9; Within-patient longitudinal (pre–post)
Prashad et al. (2023)	Non-CNS; Non-metastatic breast cancer (AJCC stages 0–III); Adults	USA	Cross-sectional patient-control study; Post-surgery, pre-adjuvant treatment	Patients: 42; Comparators: 28; Patients vs healthy controls
Quik et al. (2012)	Primary CNS/intracranial; Mixed primary intracranial tumors treated with cranial radiotherapy in adulthood; Adults	Netherlands	Cross-sectional patient subgroup study; Survivorship after cranial radiotherapy	Patients: 19; Patient subgroup comparison (GHD vs non-GHD)
Sadaghiani et al. (2019)	Primary CNS/intracranial; Resected frontal low-grade glioma (n=14) or meningioma (n=9); Adults	Norway	Cross-sectional lesion-control study; Chronic post-surgery	Patients: 23; Comparators: 13; Patient lesion subgroups vs healthy controls; Dimensional lesion analyses
Schagen et al. (2001)	Non-CNS; Breast cancer (high-risk chemotherapy groups; stage I non-chemotherapy controls); Adults	Netherlands	Cross-sectional follow-up nested in a randomized chemotherapy trial, with nonrandomized cancer controls; Survivorship (approximately 2 years post-treatment)	Patients: 33; Comparators: 14; Randomized chemotherapy-dose groups vs nonrandomized cancer controls
Schellart et al. (2011)	Mixed/other; primary and metastatic intracranial tumors: primary brain tumors (n = 5), CNS lymphoma (n = 2), and solitary brain metastases (n = 3); Adults	Netherlands	Prospective uncontrolled pre–post pilot intervention with historical healthy comparisons; Chronic survivorship; Pre/post supportive intervention	Patients: 10; Comparators: 23; Within-patient longitudinal intervention; Historical healthy comparisons
Schoenberg et al. (2024)	Non-CNS; Head and neck cancer: oropharyngeal (n=10) and nasopharyngeal (n=1); Adults	USA	Retrospective cross-sectional cohort study; Survivorship (mean 4.6 years; range 1–16 years post-treatment)	Patients: 11; Comparators: 10; Patients vs healthy controls
Siddiqui et al. (1992)	Non-CNS; Mixed non-CNS cancer: lung (n=14), tonsil (n=1), and maxillary sinus (n=1); Adults	USA	Prospective controlled cross-sectional study; Pre-treatment	Patients: 16; Comparators: 28; Patients vs healthy controls

Panel A. Study and sample characteristics (continued)

Citation	Population	Country	Design and assessment timing	Sample and comparison
Sideroff et al. (2023)	Non-CNS; Breast cancer; Adults	USA	Uncontrolled pre-post pilot intervention study; Chronic survivorship (≥1 year post-chemotherapy); Pre/post supportive intervention	Patients: 9; Within-patient longitudinal (pre-post)
Simó et al. (2018)	Non-CNS; Lung cancer: small-cell (SCLC; n=17) and non-small-cell (NSCLC; n=15); Adults	Spain	Cross-sectional three-group study (baseline analysis of a longitudinal cohort); Mixed: pre-chemotherapy and one month post-chemotherapy	Patients: 32; Comparators: 18; Patients vs patients and healthy controls
Solbakk et al. (2014)	Mixed intracranial lesion sample; Mixed focal OFC lesions: resected intracranial tumor (8 meningiomas, 1 low-grade glioma) and traumatic brain injury (n=3); Adults	Norway	Cross-sectional lesion-control study; Post-surgery/injury (at least 6 months)	Patients: 12; Comparators: 14; Mixed clinical lesion group vs healthy controls
Swainston et al. (2021)	Non-CNS; Breast cancer (primary, non-metastatic); Adults	UK	Cross-sectional; Survivorship after active treatment	Patients: 30; Comparators: 32; Patients vs healthy controls
Van Dyk et al. (2017)	Non-CNS; Breast cancer (stage 0-IIIA); Adults	USA	Cross-sectional analysis of a prospective longitudinal cohort; Long-term survivorship (approximately 5 years after diagnosis)	Patients: 62; Single survivor cohort with internal cognitive/depression subgroups
Überall et al. (1996)	Non-CNS; Childhood acute lymphoblastic leukemia without CNS involvement; mixed pediatric/adult	Germany	Cross-sectional three-group survivorship study; Long-term survivorship (mean 7.1 years post-treatment)	Patients: 13; Comparators: 13; Patient treatment groups vs healthy controls
Vasaghi Gharamaleki et al. (2022)	Non-CNS; Breast cancer (stage 0-III); Adults	Iran	Cross-sectional comparative observational study; Survivorship (at least 6 months post-chemotherapy)	Patients: 35; Comparators: 24; Patient treatment groups vs healthy controls
Wirkner et al. (2017)	Non-CNS; Breast cancer; Adults	Germany	Cross-sectional pilot with 1-week delayed recognition test; Survivorship (mean 2.83 years post-treatment)	Patients: 20; Comparators: 31; Patients vs healthy controls
Wolthuis et al. (2021)[d]	Primary CNS/intracranial; Primary brain tumors: diffuse astrocytoma grade II (n=5), oligodendroglioma grade II (n=10), and meningioma grade I (n=10); Adults	Netherlands	Prospective longitudinal observational study; Mixed: pre-surgery and 1-year post-surgery; many glioma patients also received adjuvant treatment	Patients: 25; Comparators: 24; Patient tumor groups vs matched healthy controls; Longitudinal

Panel A. Study and sample characteristics (continued)

Citation	Population	Country	Design and assessment timing	Sample and comparison
Wolthuis et al. (2022)[d]	Primary CNS/intracranial; Primary brain tumors: diffuse astrocytoma grade II (n=5), oligodendroglioma grade II (n=10), and meningioma grade I (n=10); Adults	Netherlands	Prospective longitudinal observational study; Mixed: pre-surgery and 1-year post-surgery; Many glioma patients also received adjuvant treatment	Patients: 25; Comparators: 24; Patient tumor groups vs matched healthy controls; Longitudinal
Xu et al. (2020)	Non-CNS; Primary breast cancer; Adults	China	Retrospective cross-sectional comparison; Mixed: pre-treatment, during chemotherapy and survivorship	Patients: 529; Cross-sectional patient treatment-stage groups
Yang et al. (2022)	Primary CNS/intracranial; Mixed intra-axial brain tumors: low-grade (n=14) and high-grade (n=7), with heterogeneous locations; Adults	China	Cross-sectional case-control study; Pre-surgery	Patients: 21; Comparators: 21; Patients vs healthy controls
Zhang et al. (2019)	Non-CNS; Nasopharyngeal carcinoma (stages II-IV); Adults	China	Randomized comparative trial; EEG at enrollment and 6 months after treatment	Patients: 80; Two randomized treatment groups (n=40 each)

Panel B. EEG and cognitive assessment characteristics

Citation	Recording context and paradigm	EEG approach and principal measures	Cognitive and related outcomes	Direct EEG–outcome analysis
Anderson et al. (2018)	Driving simulation with visual-search task under low and high cognitive load	ERP: Eye fixation-related potential (EFRP); P1	Behavioral task performance: Visual search task (Landolt C); Driving simulation task	Significant association reported
Anderson et al. (2019)	Visual search (spatial attention); Change detection (working memory/filtering)	ERP: N2pc; CDA	Standardized cognitive tests; Behavioral task performance: Trail Making Test (A/B); Stroop (Golden); PASAT; UFOV	No direct association tested
Anderson et al. (2020)	Visual search (spatial attention); Change detection (working memory/filtering)	ERP: N2pc; CDA	Standardized cognitive tests; Behavioral task performance: Trail Making Test A/B; Golden Stroop; PASAT; UFOV	No direct association tested
Arapova et al. (2022)	Resting EEG (waking: eyes closed/open); nocturnal sleep with REM analysis	qEEG: Absolute spectral power (0.5-19.8 Hz) in delta, theta, alpha and beta bands during eyes-closed/eyes-open waking and REM sleep; REM arousal index	Standardized cognitive tests; Clinician-rated functional measures: MoCA; MMSE; Frontal Assessment Battery; Barthel Index; Karnofsky Performance Status	No direct association tested
Berger et al. (2023)	Visual search task (spatial attention; distraction); Change detection task (working memory load/filtering)	ERP: P2; N2; P2N2; N2pc; CDA	Standardized cognitive tests; Subjective/related measures: Trail Making Test (A/B); Stroop; Symbol Digit Modalities Test; Auditory Verbal Learning Test (AVLT); Global Deficit Score (GDS); additional reported outcomes included FACT-Cog and symptom/QoL measures	Qualified/ mixed findings across outcomes
Bian et al. (2019)	Not reported	ERP: P300 amplitude and latency	Standardized cognitive tests: MMSE; MoCA	No direct association tested
Brace et al. (2019)	Auditory oddball with frequency and intensity deviants under passive-ignore and active-attend conditions	ERP: P1; mismatch negativity (MMN); P3a; P3b	Behavioral task performance: Oddball hit rate, false-alarm rate, and response time; psychometric tests were used only to screen controls	No direct association tested

Panel B. EEG and cognitive assessment characteristics (continued)

Citation	Recording context and paradigm	EEG approach and principal measures	Cognitive and related outcomes	Direct EEG–outcome analysis
Cao et al. (2017)	Resting state, awake with eyes closed	qEEG: Theta (4-8 Hz), alpha (8-13 Hz), and beta (13-20 Hz) power; categorical frontal abnormalities including alpha forward/generalization, alpha asymmetry, alpha moderation, and increased theta activity	Standardized cognitive tests; Subjective and clinician-rated mood measures: WAIS IQ; Color Trails Test (CTT); Stroop test; verbal fluency test (VFT); Figure fluency test (FFT); Beck Depression Inventory-II; Brief Psychiatric Rating Scale	Significant association reported
Caraceni et al. (1993)	Auditory oddball; mentally count 2000-Hz targets among 750-Hz standards	ERP: N1; P2; N2; P3a; P3b	Standardized cognitive tests: MMSE; verbal span; WAIS Digit Span forward/backward; Corsi spatial span forward/backward; Zazzo attention test	No direct association tested
Ch'ien et al. (1981)	Awake resting EEG with additional ictal/interictal recordings when clinically indicated	qEEG: Age-adjusted parieto-occipital background frequency; focal or bilateral delta/theta slowing; sharp waves, spikes, and ictal discharges	Standardized cognitive tests: WISC; Peabody Picture Vocabulary Test (PPVT); Other test batteries in selected long-term survivors	No direct association tested
Clavio et al. (2004)	Resting EEG; recording-state details referred to a previous methods paper	qEEG: Relative power in 2-4, 4.5-8, 8.5-12, and 12.5-32 Hz bands; composite qEEG-I based on band-power Z-scores	Standardized cognitive tests; Subjective/related measures: MMSE; Visual Search Test; Trail Making Test A/B; Weigl's Sorting Test; Buschke-Fuld Selective Reminding Test; FACT-An fatigue and anemia-related quality-of-life questionnaire	No direct association tested
Ercoli et al. (2013)	Resting state, awake with eyes closed; EEG obtained at baseline and immediate post-intervention	qEEG: Global absolute and relative delta (0.5-4 Hz), theta (4-8 Hz), alpha (8-12/13 Hz), and beta (12/13-20 Hz) power; anterior-posterior alpha gradient	Standardized cognitive tests; Subjective/related measures: PAOFI; CNS Vital Signs; HVLT-R; BVMT-R; Trail Making Test A/B; PASAT; Judgment of Line Orientation	Significant association reported

Panel B. EEG and cognitive assessment characteristics (continued)

Citation	Recording context and paradigm	EEG approach and principal measures	Cognitive and related outcomes	Direct EEG–outcome analysis
Ercoli et al. (2015)	Resting state, awake; assessed at baseline, immediately post-intervention, and 2-month follow-up	qEEG: Global absolute and relative delta (0.5-4 Hz), theta (4-8 Hz), alpha (8-13 Hz), and beta (13-20 Hz) power; anterior-posterior absolute-alpha gradient	Standardized cognitive tests; Subjective/related measures: PAOFI; RAVLT; BVMT-R; verbal fluency; PASAT; Trail Making Test A/B; CNS Vital Signs; WTAR estimated IQ	Significant association reported
Gan et al. (2019)	Implicit emotion processing (arithmetic judgment over emotional images) and explicit emotion processing (negative/neutral valence judgment)	ERP: N1; P2; N2; P3	Standardized cognitive tests; Behavioral task performance; Subjective/related measures: MoCA; implicit arithmetic-over-emotional-picture task; explicit emotional-valence judgment task; Emotion Regulation Questionnaire (ERQ-CRV)	Significant association reported
Heukrodt et al. (1988)	Auditory oddball with two rare-tone counting runs and one ignore-tone/video-game counting run	ERP: P3/P300 latency and amplitude	Standardized cognitive tests: WAIS or WISC-R; Wide Range Achievement Test (WRAT); age-appropriate Halstead-Reitan Neuropsychological Battery	Significant association reported
Hung et al. (2020)	Visual SART with block-end experience sampling of on-task versus mind-wandering states	ERP: P300	Standardized cognitive tests; Behavioral task performance; Subjective/related measures: SART with experience sampling; HVLT-R; computerized Stroop; Trail Making Test A/B; COWA (FAS); Animal Naming; FACT-Cog	No direct association tested
Hunter et al. (2014)	Eyes-closed resting state (minimum 10-minute recording)	qEEG: Global and electrode-level absolute and relative power: delta (0.5–4 Hz), theta (4–8 Hz), alpha (8–13 Hz), beta (13–20 Hz)	Subjective cognition: Patient Assessment of Own Functioning Inventory (PAOFI); WTAR-estimated IQ available from parent studies	Significant association reported
Järvelä et al. (2011)	Passive auditory oddball using Finnish vowels (/o/ standard, /e/ deviant) while participants watched a silent video	ERP: MMN; P3a	Standardized cognitive tests: Age-appropriate BSID-II Mental Scale, WPPSI-R, WISC-III, or WAIS-R	Significant association reported

Panel B. EEG and cognitive assessment characteristics (continued)

Citation	Recording context and paradigm	EEG approach and principal measures	Cognitive and related outcomes	Direct EEG–outcome analysis
Kam et al. (2016)	Visual SART with attentional-state probes; separate eyes-closed resting recording	ERP: P300/P3 amplitude and peak latency; qEEG: Absolute power in low alpha (8–10 Hz), high alpha (10–12 Hz), low beta (12–20 Hz), and high beta (20–30 Hz), summarized across frontal, central, parietal, and temporal regions	Behavioral task performance: Sustained Attention to Response Task (SART) with repeated on-task/mind-wandering experience sampling; no conventional neuropsychological battery reported	Tested; no significant association
Krawutschke et al. (2025)	Visual oddball/valence-judgment task with neutral standards and positive/negative, low/high-arousal IAPS deviants	ERP: P300 peak amplitude at Pz (250–800 ms)	Behavioral task performance; Subjective/related measures: Visual emotional oddball/valence-judgment task using IAPS pictures; reaction time; PHQ-9	No direct association tested
Kreukels et al. (2005)[a]	Visual AFM task manipulating stimulus discriminability and stimulus-response compatibility; response complexity was omitted from final analyses because of high error rates	ERP: P3/P300 peak latency and amplitude at Pz	Behavioral task performance; Subjective/related measures: Additive Factors Method (AFM) reaction-time task; Semistructured interview on memory, concentration, thinking and language complaints	Significant association reported
Kreukels et al. (2006)[a]	Visual AFM task manipulating stimulus discriminability and stimulus-response compatibility; response complexity was omitted from final analyses because of high error rates	ERP: P3/P300 peak latency and amplitude at Pz	Behavioral task performance; Subjective/related measures: Additive Factors Method (AFM) reaction-time task; Semistructured interview on memory, concentration, thinking and language complaints	Significant association reported
Kreukels et al. (2008a)[a]	Auditory oddball: mentally count rare 2000-Hz tones among frequent 1000-Hz tones and report the count afterward	ERP: N1 and P3/P300 latency, amplitude and scalp-field peak/trough coordinates	Behavioral task performance: Mental count accuracy during auditory oddball task	No direct association tested

Panel B. EEG and cognitive assessment characteristics (continued)

Citation	Recording context and paradigm	EEG approach and principal measures	Cognitive and related outcomes	Direct EEG–outcome analysis
Kreukels et al. (2008b)[a]	Visual information-processing/AFM task using double-arrowhead stimuli	ERP: P3/P300 peak latency and amplitude at Pz	Standardized cognitive tests; Behavioral task performance: T1: Trail Making A/B; WAIS Digit Symbol; Stroop; Eriksen task; Working-Memory Updating; Dutch CVLT; WMS Visual Reproduction; AFM task; Word Fluency; FePsy Finger Tapping. T2: AFM-like reaction-time/error task during EEG.	Significant association reported
Kroczka et al. (2022)	Auditory oddball with two contrasting tones; press a counter for deviant stimuli	ERP: P300 latency and amplitude at Pz; button-press reaction time	Behavioral task performance: Oddball target-detection accuracy and response time	No direct association tested
Kuleva et al. (2022)	Resting state with eyes closed for 2–3 minutes; spectral coherence analysis	qEEG: Pairwise EEG coherence in delta (0.4–3.9 Hz), theta1 (4.3–5.5), theta2 (5.6–7.3), alpha1 (7.8–8.6), alpha2 (9–10.2), alpha3 (10.5–12.5), and beta1 (12.9–20.7 Hz)	Behavioral task performance; Subjective/related measures: Eyetracker–Attention–Memory (EAM) visual memorization task; Resting State Questionnaire (ReSQ: available for 12/14 patients)	No direct association tested
Li et al. (2022)	Pain-empathy picture paradigm crossing judgment task (pain vs laterality) with stimulus type (painful vs neutral)	ERP: N1 (50–150 ms) and P2 (150–200 ms) amplitudes at Fz, FCz, and Cz	Standardized cognitive tests; Behavioral task performance; Subjective/related measures: MMSE; Digit Span Forward and Backward; Verbal fluency test; Chinese Interpersonal Reactivity Index; Pain-empathy task accuracy and response time	Significant association reported
Lin et al. (2022)	Auditory oddball cognitive-potential test; procedural details beyond Cz recording were not reported	ERP: P300 amplitude and latency at Cz	Subjective/related measures; FACT-Cog and Cancer Fatigue Scale cognitive-fatigue subscale; MMSE used only for eligibility and baseline screening	No direct association tested

Panel B. EEG and cognitive assessment characteristics (continued)

Citation	Recording context and paradigm	EEG approach and principal measures	Cognitive and related outcomes	Direct EEG–outcome analysis
Lähteenmäki (1999)[b]	Modified Sternberg auditory memory-search task with separate encoding and probe-retrieval phases; five-minute eyes-closed resting EEG	qEEG: Task-related alpha ERD/ERS (8–10 and 10–12 Hz); resting absolute and relative spectral power (0–30 Hz)	Behavioral task performance: Modified Sternberg auditory memory-search task (four-word encoding and probe recognition)	No direct association tested
Lähteenmäki et al. (2001)[b]	Auditory oddball: passive frequency-change condition while watching a silent cartoon for MMN/P3a, followed by an active deviant-counting condition for P300	ERP: P300 (P3b) peak amplitude/latency; MMN and P3a area, amplitude, and latency parameters	Academic performance measures: Most recent school-report marks (mathematics, oral expression/writing, and first foreign language); No formal neuropsychological test battery	No direct association tested
Mathan et al. (2010)	Time-constrained reading under high versus low workload, with simple acknowledgement or two-digit-addition interruptions	qEEG: Spectral power: theta (4–8 Hz), alpha (8–12 Hz), beta (12–30 Hz), and gamma (30–40 Hz)	Behavioral task performance; Subjective/related measures: Cognitive symptoms checklist; reading/interruption workload task; Modified Cooper–Harper subjective workload rating	No direct association tested
Melara et al. (2023)[c]	ANT-R flanker task with no, double, valid, and invalid spatial cues and congruent/incongruent targets	ERP: P1, N1, P3, and slow wave (800–1,000 ms)	Standardized cognitive tests; Behavioral task performance; Subjective/related measures: Revised Attention Network Test (ANT-R; reaction time and accuracy); FACT-Cog; WRAT-4	No direct association tested
Melara et al. (2025)[c]	Auditory paired-click task without behavioral response and ANT-R attention task	qEEG: Aperiodic 1/f fractal slope (1–100 Hz) as an index of neural noise	Standardized cognitive tests; Behavioral task performance; Subjective/related measures: ANT-R (reaction time and accuracy); FACT-Cog; WRAT-4	Significant association reported

Panel B. EEG and cognitive assessment characteristics (continued)

Citation	Recording context and paradigm	EEG approach and principal measures	Cognitive and related outcomes	Direct EEG–outcome analysis
Moore et al. (1992)	Auditory oddball: participants relaxed during frequent tones and counted rare tones	ERP: P1, N1, P2, and P300	Standardized cognitive tests; Behavioral task performance: WISC-R or WAIS; Academic achievement tests (WRAT/PIAT); Verbal and nonverbal Selective Reminding; Trail Making; Finger Tapping; Grooved Pegboard; Beery VMI; WISC-R Freedom from Distractibility quotient; fixed/random-ISI reaction-time task	Significant association reported
Moore et al. (2014)	EEG recorded at rest and immediately after WAIS-III processing-speed and sustained elbow-flexion tasks	qEEG: Absolute total-spectrum EEG power (FFT; 0.1–100 Hz), principally from the left central region	Standardized cognitive tests; Subjective/related measures: WAIS-III Processing Speed Index (Symbol Search and Digit Symbol-Coding); Brief Mental Fatigue Questionnaire; Brief Fatigue Inventory	Qualified: significant in pooled sample only
Münte et al. (2004)	Visual target detection using automatic parallel search and effortful serial search with set sizes 4, 8, and 12	ERP: P1; N1; P3	Standardized cognitive tests; Behavioral task performance: d2 Test of Attention; WAIS Digit Span Forward; Stroop test; Verbal Learning and Memory Test (VLMT); Parallel and serial visual-search task performance	Significant association reported
Olbrich et al. (1986)	Auditory oddball (800-Hz frequent tones; 1400-Hz rare tones); participants counted rare tones	ERP: N1; P2; N2; P3	Clinician-rated cognitive classification: Assessment of Symptoms in Gerontopsychiatry (AGP; modified AMDP scale)	Significant association reported
Pace et al. (1994)	Visual checkerboard oddball with mental target counting; the report did not state whether the spectral EEG epochs were recorded at rest or during the task	ERP: N2; P300; qEEG: Relative alpha, delta, theta, and beta frequency-band percentages and cerebral mapping	Standardized cognitive tests: Mini-Mental State Examination (MMSE)	No direct association tested

Panel B. EEG and cognitive assessment characteristics (continued)

Citation	Recording context and paradigm	EEG approach and principal measures	Cognitive and related outcomes	Direct EEG–outcome analysis
Parageorgiou et al. (2000)	Warning-tone-cued digit-span task; ERPs recorded during the 1-s interval before digit presentation	ERP: P50; N100; P300; N400	Behavioral task performance: Computerized Wechsler Digit Span task with forward or reverse recall cued by warning-tone frequency. Subjective/related measures: Zung Self-Rating Depression Scale and Spielberger Anxiety Inventory	No direct association tested
Pietrangeli et al. (1990)	Auditory oddball (frequent 100-Hz and rare 3000-Hz tones); participants counted rare tones	ERP: P3	No formal cognitive test was reported; participants counted rare tones during the auditory oddball task, but performance was not reported	No direct association tested
Prashad et al. (2023)	Visuospatial Attention Network Task with no, double, valid, and invalid cue conditions and congruent/incongruent flankers	qEEG: Frontal alpha asymmetry from induced low-alpha (8–10 Hz) and high-alpha (11–13 Hz) power across early, middle, and late post-stimulus epochs	Standardized cognitive tests; Behavioral task performance: Visuospatial Attention Network Task (reaction time and accuracy); WRAT-4 as a proxy for cognitive reserve	No direct association tested
Quik et al. (2012)	Go/no-go selective-attention/selection-potential task	ERP: N2b, P300, and lateralized readiness potential (LRP)	Standardized cognitive tests; Behavioral task performance: WAIS-III Digit Span, Digit Symbol and Similarities; 15-Word Test; Rey–Osterrieth Complex Figure; Stroop; Trail Making A/B; Brixton; verbal fluency; Dutch Adult Reading Test; Go/no-go selective-attention task during EEG	No direct association tested
Sadaghiani et al. (2019)	Eyes-closed resting state	qEEG: Relative global phase-locking value (PLV) modulation, quantified as range and SD, in high-alpha (10–12 Hz) and control frequency bands; alpha-power control analyses	Standardized cognitive tests: D-KEFS Trail Making Test, primarily Number–Letter Switching (age-standardized score)	Qualified: patient association trend-level

Panel B. EEG and cognitive assessment characteristics (continued)

Citation	Recording context and paradigm	EEG approach and principal measures	Cognitive and related outcomes	Direct EEG–outcome analysis
Schagen et al. (2001)	Eyes-closed/eyes-open resting EEG and auditory oddball target-counting task	ERP: P300 latency; qEEG: Alpha peak frequency, eyes-open alpha blocking, and interhemispheric alpha-frequency asymmetry	Standardized cognitive tests; Subjective/related measures: Thirteen-test neuropsychological battery, domain scores and total deviant-test count; Interview on daily cognitive problems; EORTC QLQ-C30; Hopkins Symptom Checklist	Significant association reported
Schellart et al. (2011)	Visual checkerboard oddball with silent counting of rare stimuli	ERP: Visual C1, N200, P3a, and P3b amplitude and latency; P3b amplitude was the principal treatment-sensitive measure	Standardized cognitive tests; Informant-rated cognition: Informant Questionnaire on Cognitive Decline in the Elderly (IQCODE); Number Connection Test; Continuous Reaction Time Test; oddball counting during EEG	Tested, no significant association
Schoenberg et al. (2024)	Visual cognitive and emotional Go/Nogo paradigms using letters and neutral, positive, and pain-related words	ERP: C1; P1; N1; N2; P3; N4	Behavioral task performance; Subjective/related measures: Cognitive and emotional visual Go/Nogo tasks; BRIEF-A; PROMIS-29; VHNSS/GSS; Neurotoxicity Rating Scale	Significant association reported
Siddiqui et al. (1992)	Auditory oddball target counting (1-kHz frequent and 2-kHz rare tones)	ERP: P300; qEEG: Alpha frequency reported descriptively	Behavioral task performance: Target-tone counting accuracy during the auditory oddball task; No formal neuropsychological testing	No direct association tested
Sideroff et al. (2023)	Eyes-open and eyes-closed resting qEEG; 18 sessions of theta/high-beta inhibition and low-beta/SMR reinforcement	qEEG: Spectral magnitude in delta, theta, alpha, beta, and gamma bands; abnormality defined as ≥2 SD from normative values	Standardized cognitive tests; Subjective/related measures: Auditory Consonant Trigrams; Trail Making Test A/B; Stroop; Digit Span; Continuous Performance Test; Brief Symptom Inventory; Resilience Assessment Profile	No direct association tested

Panel B. EEG and cognitive assessment characteristics (continued)

Citation	Recording context and paradigm	EEG approach and principal measures	Cognitive and related outcomes	Direct EEG–outcome analysis
Simó et al. (2018)	Modified Eriksen flanker plus stop-signal task assessing response and conflict monitoring	ERP: ERN; CRN; N1; P2; N2; P300	Standardized cognitive tests; Behavioral task performance; Subjective/related measures: MDRS-2; WAIS-III subtests; RAVLT; WMS-III Logical Memory; Rey–Osterrieth Complex Figure; Boston Naming Test; phonemic/semantic fluency; Trail Making Test A/B; flanker/stop-signal task; BDI	No direct association tested
Solbakk et al. (2014)	Visual stop-signal task with congruent/incongruent go trials and successful/failed stop trials	ERP: Go P3; stop N2/P3; ERN; Pe; qEEG: Task-related theta and beta power	Standardized cognitive tests; Behavioral task performance; Clinician-rated global outcome: Visual stop-signal task; WASI four-subtest full-scale IQ; Extended Glasgow Outcome Scale	No direct association tested
Swainston et al. (2021)	Modified letter flanker task with congruent and incongruent trials	ERP: ERN; CRN; delta-ERN; early and late Pe	Behavioral task performance; Subjective/related measures: Modified Eriksen flanker task; FACT-Cog v3; OSPAN working-memory task (supplementary analysis)	No direct association tested
Van Dyk et al. (2017)	Resting state, eyes closed	qEEG: Absolute/relative delta, theta, alpha, and beta power; global/prefrontal/anterior/posterior summaries; anterior-posterior gradients; cordance	Standardized cognitive tests; Subjective/related measures: FACT-Cog v3; CVLT-II; WMS-IV Logical Memory; BVMT-R; Rey-Osterrieth Complex Figure; WAIS-IV subtests; Trail Making Test; PASAT; verbal fluency; Grooved Pegboard; WTAR	Significant association reported
Überall et al. (1996)	Visual checkerboard-reversal oddball with button response to rare target pattern	ERP: P300 latency, amplitude, and scalp topography	Standardized cognitive tests: WISC-R/WAIS-R; Culture Fair Test; d2 concentration test; Recurring Figures Test	No direct association tested

Panel B. EEG and cognitive assessment characteristics (continued)

Citation	Recording context and paradigm	EEG approach and principal measures	Cognitive and related outcomes	Direct EEG–outcome analysis
Vasaghi Gharamaleki et al. (2022)	Resting state and performance of each participant's Area Needing Improvement task; separate HEG was recorded at FP1 before, during, and after the task	qEEG: Alpha and beta-1 absolute power; Z-scored FFT coherence; phase lag; current source density at 10 and 15 Hz in dorsal and ventral attention networks	Standardized cognitive tests: Addenbrooke's Cognitive Examination; Cambridge Brain Sciences 12-task online battery; Each participant's lowest-scoring task defined the Area Needing Improvement task	No direct association tested
Wirkner et al. (2017)	Free viewing/incidental encoding of unpleasant, pleasant, and neutral pictures; recognition tested 1 week later	ERP: Late positive potential (LPP; 400-800 and 800-1200 ms)	Standardized cognitive tests; Behavioral task performance; Subjective/related measures: TAP alertness/divided attention/go-no-go/working memory; WMS-R Digit Span and Logical Memory; VLMT; Picture-recognition performance 1 week after incidental encoding; Subjective concentration/memory ratings	Qualified/ mixed findings across outcomes
Wolthuis et al. (2021)[d]	Eyes-closed resting state (at least 5 min; extended to 10 min if needed)	qEEG: Theta- and alpha-band phase lag index (PLI); weighted-network measures (relative clustering, relative path length, small-world index); minimum-spanning-tree measures (degree, eccentricity, diameter, hierarchy, betweenness centrality, leaf fraction).	Standardized cognitive tests: Object naming; action naming in sentence context; Shortened Token Test; category and letter fluency; reading/writing; DIMA repetition, semantic odd-picture-out, sentence completion and sentence judgment; domain z-scores based on normative data.	Significant association reported
Wolthuis et al. (2022)[d]	Eyes-closed resting state (at least 5 min; extended to 10 min if needed)	qEEG: Visual grading of abnormal delta/theta slowing; relative delta and theta power over the whole brain, tumor-affected hemisphere and tumor-affected area.	Standardized cognitive tests: Object naming; Action naming in sentence context; Category and letter fluency; Reading/writing; DIMA repetition, semantic odd-picture-out and sentence completion; domain z-scores based on normative data.	Significant association reported

Panel B. EEG and cognitive assessment characteristics (continued)

Citation	Recording context and paradigm	EEG approach and principal measures	Cognitive and related outcomes	Direct EEG–outcome analysis
Xu et al. (2020)	Auditory oddball with 1,000-Hz standard and 2,000-Hz target tones; target probabilities 0.10 and 0.15.	ERP: P300	No independent neuropsychological, behavioral or self-report cognitive assessment.	No direct association tested
Yang et al. (2022)	Passive viewing of Chinese characters, neutral faces and common objects	ERP: P1/N1, N170/VPP, P2 and P3a; qEEG: Task-activated source power and 9-30-Hz source-level debiased weighted phase-lag index (dwPLI) connectivity.	Subjective/related measures: Study-specific self-rating followed by clinical interview; No behavioral outcome from the passive task	No direct association tested
Zhang et al. (2019)	Auditory target/non-target paradigm; target stimulus repeated 30 times	ERP: P300	Standardized cognitive tests: Mini-Mental State Examination (MMSE); Auditory P300 was treated as an objective cognitive indicator.	No direct association tested

Notes

Overlapping cohorts. Counts refer to articles rather than independent cohorts. Superscripts indicate known or probable overlap: a, Kreukels et al. (2005, 2006, 2008a, 2008b); b, Lähteenmäki et al. (1999, 2001); c, Melara et al. (2023, 2025); and d, Wolthuis et al. (2021, 2022).

Sample sizes. Counts refer to the principal EEG analytic sample. Analysis-specific denominators are shown when they differed substantially between paradigms or time points.

Direct EEG–outcome analysis. Direct associations required a statistical relationship between an EEG measure and a cognitive, subjective or symptom-related, clinician-rated, or behavioral outcome within a cancer sample or longitudinal patient analysis. Pooled patient/control findings, trend-level associations, and mixed findings were classified as qualified. Task-related EEG modulation, group differences, and parallel changes alone were not classified as direct associations. Of the 60 articles, 21 reported a significant direct association, 4 reported qualified or mixed findings, 2 tested but did not find a significant association, and 33 did not test a direct association.

Abbreviations. ALL, acute lymphoblastic leukemia; AML, acute myeloid leukemia; ANT-R, revised Attention Network Test; CDA, contralateral delay activity; CLL, chronic lymphocytic leukemia; CNS, central nervous system; dwPLI, debiased weighted phase lag index; EEG, electroencephalography; ERP, event-related potential; FACT-Cog, Functional Assessment of Cancer Therapy–Cognitive Function; MMN, mismatch negativity; NHL, non-Hodgkin lymphoma; PAOFI, Patient Assessment of Own Functioning Inventory; qEEG, quantitative EEG; RCT, randomized controlled trial; SART, Sustained Attention to Response Task.
